# ALKBH5 facilitates CYP1B1 mRNA degradation via m6A demethylation to alleviate MSC senescence and osteoarthritis progression

**DOI:** 10.1038/s12276-023-01059-0

**Published:** 2023-08-01

**Authors:** Guiwen Ye, Jinteng Li, Wenhui Yu, Zhongyu Xie, Guan Zheng, Wenjie Liu, Shan Wang, Qian Cao, Jiajie Lin, Zepeng Su, Dateng Li, Yunshu Che, Shuai Fan, Peng Wang, Yanfeng Wu, Huiyong Shen

**Affiliations:** 1 Department of Orthopedics, The Eighth Affiliated Hospital, Sun Yat-sen University, Shenzhen, 518033 PR China; 2 Center for Biotherapy, The Eighth Affiliated Hospital, Sun Yat-sen University, Shenzhen, 518033 PR China; 3 Department of Statistical Science, Southern Methodist University, Dallas, TX USA

**Keywords:** Senescence, Mesenchymal stem cells, Diseases

## Abstract

Improving health and delaying aging is the focus of medical research. Previous studies have shown that mesenchymal stem cell (MSC) senescence is closely related to organic aging and the development of aging-related diseases such as osteoarthritis (OA). m6A is a common RNA modification that plays an important role in regulating cell biological functions, and ALKBH5 is one of the key m6A demethylases. However, the role of m6A and ALKBH5 in MSC senescence is still unclear. Here, we found that the m6A level was enhanced and ALKBH5 expression was decreased in aging MSCs induced by multiple replications, H_2_O_2_ stimulation or UV irradiation. Downregulation of ALKBH5 expression facilitated MSC senescence by enhancing the stability of CYP1B1 mRNA and inducing mitochondrial dysfunction. In addition, IGF2BP1 was identified as the m6A reader restraining the degradation of m6A-modified CYP1B1 mRNA. Furthermore, Alkbh5 knockout in MSCs aggravated spontaneous OA in mice, and overexpression of Alkbh5 improved the efficacy of MSCs in OA. Overall, this study revealed a novel mechanism of m6A in MSC senescence and identified promising targets to protect against aging and OA.

## Osteoarthritis: Age-related stem cell modifications point to new treatments

The age-related modification of adult stem cells, known as mesenchymal stem cells (MSCs), could be targeted to alleviate the progression of osteoarthritis. The deterioration of cellular functions due to aging (or ‘senescence’) of MSCs is linked to the development of diseases including osteoarthritis. A common RNA modification in mammal cells, m6A, plays vital roles in cellular functioning. Guiwen Ye at Sun Yat-sen University in Shenzhen, China, and co-workers used patient cell lines and mouse models to show that m6A levels are enhanced in aging MSCs. This increase is due to reduced expression of a key protein called ALKBH5 and modifies the behavior of a molecule found in MSC mitochondria called CYP1B1. This leads to mitochondrial dysfunction and MSC senescence. Knocking out the *Alkbh5* gene in mouse MSCs aggravated osteoarthritis, while boosting *Alkbh5* expression in MSCs reduced osteoarthritis progression.

## Introduction

Aging is a critical factor affecting human health and is closely related to the occurrence of degenerative diseases, such as osteoarthritis (OA) and osteoporosis (OP)^1^. Studies have found that cellular senescence is an important cause of organism aging^2^. Due to multiple replications, oxidative stress or radiation damage, cells enter a state of senescence characterized by permanent cell cycle arrest and changes in cell morphology^3^. In addition, cellular dysfunction and senescence-associated secretory phenotypes (SASPs) are observed in senescent cells^3^. Previous studies have shown that stem cell senescence is an important cause of organism aging. For example, decelerated senescence of mesenchymal precursor cells alleviated liver aging and extended lifespan^4^. However, more research on this topic is needed.

Mesenchymal stem cells (MSCs) are stem cells that are widespread in bone marrow, umbilical cord blood, synovium, ligaments and other tissues^5^. MSCs possess a strong immunoregulatory capability and the potential to differentiate into osteoblasts, adipoblasts and chondroblasts^5^. MSCs play an important role in organism growth and development, tissue damage repair and immune inflammatory homeostasis^6^. In addition, MSCs are widely used in the treatment of immune-related diseases and tissue engineering repair^6^. The senescence of MSCs causes functional abnormalities and may be involved in the pathogenesis of degenerative diseases^7–9^. It is important to elucidate the mechanism of MSC senescence.

RNA modification is a crucial epigenetic modification, among which m6A modification is the most abundant^10^. m6A modification is usually present in the consensus RNA motif of RRACH (R = A or G; H = A, U, or C) and strongly affects gene expression and regulation of cell function^10^. m6A modification is coregulated by “writers”, such as METTL3 and METTL14, and “erasers”, including FTO and ALKBH5. In addition, m6A “readers”, such as YTHDC1/2 and IGF2BP1/2/3, recognize the m6A modification and modulate the characteristics of RNA^11^. Studies have shown that m6A participates in the regulation of cellular senescence. For instance, Shafik revealed that m6A plays an important role in brain aging and neurodegenerative disease^12^. ALKBH5 is a m6A demethylase that participates in metabolic processes, stability and transport of RNA and various biological processes^13^. Yu showed that ALKBH5 played an important role in protecting cells from DNA damage and apoptosis^14^. However, the role of m6A and ALKBH5 in MSC senescence and organism aging requires further exploration.

CYP1B1 is a member of the cytochrome P450 superfamily possessing monooxygenase activity^15^. This molecule is mainly located in mitochondria and participates in the metabolism of physiological compounds, including estrogen, arachidonic acid, and melatonin^16^. Previously, CYP1B1 was reported to be involved in mitochondrial function and reactive oxygen species (ROS) production, two important factors of aging^17^. However, whether CYP1B1 plays a role in cellular senescence has not been reported.

In this study, we explored the role of m6A modification in MSC senescence in three models of cellular senescence and identified an important role of ALKBH5 in alleviating senescence. In addition, we found that CYP1B1 was the key downstream target of ALKBH5 and uncovered the related m6A reader IGF2BP1. Furthermore, we revealed that knockout of Alkbh5 in MSCs intensified OA in mice and that Alkbh5 helped improve the efficacy of MSC infusion on OA, providing new insights for OA therapy.

## Materials and methods

### Ethics approval

This study was approved by the Ethics Committee of The Eighth Affiliated Hospital, Sun Yat-sen University, Shenzhen, China. We confirmed that all experiments conformed to all relevant regulatory standards. Written informed consent about the experimental requirements and potential risks was provided by all subjects.

### Isolation and culture of MSCs

Bone marrow was extracted from healthy donors, and MSCs were isolated and cultured as described previously^18^. Briefly, MSCs were isolated by density gradient centrifugation at 2500 × *g* for 30 min. Then, the MSCs were collected and cultured in Dulbecco’s modified Eagle’s medium (DMEM, Gibco, 11885-076) containing 10% fetal bovine serum (FBS, Zhejiang Tianhang Biotechnology, 11011-8611). Three days later, the medium was replaced to remove the suspended cells. In addition, when the cells reached 80–90% confluence, they were digested and reseeded in two new culture flasks. MSCs were used at passage 4 in most of the experiments unless otherwise noted.

### Identification of MSCs

The surface markers and differentiation potential of MSCs were detected as previously described^19^. Briefly, MSCs were incubated with antibodies against CD29, CD44, CD105, CD45, CD14 and HLA–DR (BD Bioscience). Then, the cells were detected by flow cytometry (BD Bioscience, BD Influx) and analyzed by FlowJo V10 software. In addition, MSCs were cultured with osteogenic, chondrogenic or adipogenic induction medium and stained with oil red O, alizarin red S or Alcian blue.

### Senescence induction

For H_2_O_2_ treatment, 200 µM H_2_O_2_ was added to the MSCs, and the medium was replaced in 2 h. For ultraviolet (UV) induction, MSCs were covered with a thin layer of phosphate-buffered saline (PBS) and treated with UV irradiation (100 mJ/cm^2^) by using a UVB lamp.

### SA-β-Gal staining and β-Gal enzymatic assay

The Senescence-associated β-gal Assay Kit (Beyotime Biotechnology, C0602) was used, and SA-β-gal staining was conducted according to the manufacturer’s instructions. For MSCs seeded in culture plates, the medium was removed, and the cells were fixed with the provided fixing solution per well for 15 min at room temperature. Then, the cells were washed twice with PBS and incubated with freshly prepared SA-β-gal staining solution at 37 °C for 16–18 h with low CO_2_ and in the dark. For tissue section staining, mouse knee joints were fixed in 10% neutral formalin for 12 h and decalcified in rapid decalcification solution for 2 days at 4 °C. Then, the joints were embedded in optimal cutting temperature compound, and sections with a thickness of 8 µm were made. The frozen sections were fixed with fixing solution for 15 min and washed with PBS 3 times for 5 min each time. Then, the sections were stained with SA-β-gal staining solution in an incubator at 37 °C for 24 h with low CO_2_ and in the dark. Finally, the sections were counterstained with eosin and sealed with neutral balsam. Senescent cells were observed under a light microscope and evaluated by counting the number of blue-stained cells. The activity of β-gal was detected by using a Human β-gal ELISA Kit (ZCIBIO, ZC-32216) according to the manufacturer’s instructions.

### Western blot

Western blotting was performed as previously described^18^. Briefly, cells were lysed with RIPA buffer containing protease and phosphatase inhibitors for 30 min on ice, and the lysates were centrifuged at 14,000 rpm for 30 min at 4 °C. The protein concentrations were measured by using a BCA Protein Assay Kit (CWBIO, CW0014S) according to the instructions provided. A 5× sodium dodecyl sulfate loading buffer was added to the protein lysates, and the mixtures were boiled for 5 min. Then, the proteins were separated via polyacrylamide gel electrophoresis and transferred to polyvinylidene fluoride membranes (Millipore, IPVH0010). The membranes were blocked with 5% nonfat milk for 1 h and incubated with primary antibodies at 4 °C overnight. Then, the membranes were washed with Tris-buffered saline-Tween (TBST) solution and incubated with HRP-conjugated secondary antibodies (1:3,000, Boster, BA1050 & BA1054) for 1 h. Finally, the membranes were washed with TBST solution and detected by using Immobilon Western Chemiluminescent HRP Substrate (Millipore WBKLS0500). The details of the primary antibodies are provided in the supplementary materials.

### Cell cycle detection

The cell cycle of MSCs was detected by flow cytometry using PI/RNase Staining Buffer (BD Bioscience, 550825). Briefly, MSCs were collected and fixed in 70% cold ethanol overnight at 4 °C. Then, the MSCs were incubated with PI (5 μg/ml) and RNase A (1 mg/ml) in 500 μl of PBS at 37 °C in the dark. Thirty minutes later, flow cytometry was performed, and the data were analyzed using FlowJo V10.

### m6A dot blots

Total RNA was extracted, and 100 ng of RNA was spotted onto a nylon membrane (Sigma-Aldrich, GERPN1210B). Then, the membranes were UV-crosslinked and blocked with PBST (PBS with 0.1% Tween 20) containing 5% nonfat milk for 1 h. After that, the membranes were incubated with m6A methylation antibody (Synaptic Systems, 202003, 1:1000) at 4 °C overnight. Then, the membranes were washed with PBST and incubated with HRP-conjugated secondary antibodies (1:3,000, Boster, BA1054) for 1 h at room temperature. The membranes were washed with PBST and detected by using Immobilon Western Chemiluminescent HRP Substrate (Millipore WBKLS0500).

The same amount of RNA was spotted on another nylon membrane, and UV crosslinking was performed. Then, the membranes were stained with 0.02% methylene blue solution (pH 5.2) for 1 h. The membranes were washed with ribonuclease-free water for 2 h, and the results were captured by a camera.

### RNA extraction and quantitative real-time PCR (qPCR)

Total RNA was extracted by using TRIzol solution (TaKaRa, 9109) according to the manufacturer’s instructions. Then, the RNA was reverse transcribed into cDNA on a PCR amplifier by using a PrimeScript RT Reagent Kit (TaKaRa, RR037A). Then, qPCR was performed by using TB Green Premix Ex Taq II (TaKaRa, RR820A) according to the manufacturer’s directions, and gene expression was measured by using a real-time fluorescence quantitative PCR system (Applied Biosystems, 7500). GAPDH served as the reference gene, and the relative gene expression was determined with the 2^−ΔΔCt^ method. The relevant primers are listed in Supplementary Table 1.

### RNA interference and transfection

Small interfering RNAs (siRNAs) were designed and produced by GenePharma (Suzhou, China). When the cells reached 70–90% confluence, transfections were performed by using Lipofectamine RNAiMAX (Thermo Fisher, 13778150) according to the manufacturer’s instructions. The interference efficiency was detected by qPCR after 48 h or by western blotting after 72 h. The siRNA sequences used in this study are provided in Supplementary Table 2.

### Lentivirus construction and infection

Knockdown lentiviruses of ALKBH5 and METTL3 and overexpression lentiviruses of wild-type (WT) ALKBH5, mutant ALKBH5 and WT CYP1B1 were purchased from Obio (Shanghai, China). The cells were infected with the lentivirus at a multiplicity of infection (MOI) of 25 using polybrene (5 mg/ml). The infection medium was replaced after 24 h, and the infection efficiency was detected by qPCR after 48 h or by western blotting after 72 h.

### MeRIP-Seq and RNA-Seq

Total RNA was extracted using TRIzol reagent following the manufacturer’s procedure. The total RNA quality and quantity were analyzed using a Bioanalyzer 2100 and RNA 6000 Nano LabChip Kit (Agilent, 5067–1511) with RIN > 7.0. Approximately more than 200 µg of total RNA was subjected to isolation of poly(A) mRNA with poly-T oligo-attached magnetic beads. Following purification, the poly(A) mRNA fractions were fragmented into ~100-nt oligonucleotides using divalent cations under an elevated temperature. Then, the cleaved RNA fragments were incubated for 2 h at 4 °C with m6A methylation antibody (Synaptic Systems, 202003) in IP buffer (50 mM Tris-HCl, 750 mM NaCl and 0.5% Igepal CA-630) with BSA (0.5 μg/μl). The mixture was then incubated with Protein A beads and eluted with elution buffer (1× IP buffer and 6.7 mM m6A). Eluted RNA was precipitated with 75% ethanol. Eluted m6A-containing fragments (IP) and untreated input control fragments were converted to a final cDNA library in accordance with strand-specific library preparation by the dUTP method. The average insert size for the paired-end libraries was ~100 ± 50 bp. Then, we performed paired-end 2 × 150 bp sequencing on an Illumina Novaseq™ 6000 platform at LC-BIO Biotech, Ltd. (Hangzhou, China), following the vendor’s recommended protocol.

### MeRIP-qPCR

An EZ-Magna RIP Kit (Merck Millipore, 17–701) was used. Briefly, total RNA of different groups of MSCs was extracted and fragmented. The A/G magnetic beads and m6A methylated antibody were premixed and incubated with the RNA fragments. Then, the RNA fragments carrying m6A modification were enriched and purified. The obtained RNA and the input RNA were reverse transcribed into cDNA, and the relative expression of the target gene was detected by qPCR. The relative abundance of m6A modification in the target gene RNA was expressed as input%.

### Cellular ROS detection

The levels of cellular ROS were measured with the fluorescent probe DCFH-DA (Beyotime, S0033). Briefly, MSCs were stained with 10 µmol DCFH-DA at 37 °C in an incubator protected from light for 20 min. Then, the cells were washed with serum-free medium, and the fluorescence of DCF was detected by flow cytometry (BD Bioscience, BD Influx). The fluorescence intensity (MFI) of DCF was analyzed by FlowJo V10 software.

### Mitochondrial membrane potential detection

Mitochondrial membrane potential (∆Ψm) was detected with JC-1 probes (Beyotime, C2006). JC-1 probes form polymers and emit red fluorescence with a high ∆Ψm, while they remain as monomers and emit green fluorescence with a low ∆Ψm. MSCs were incubated with JC-1 solution and washed according to the manufacturer’s instructions. Finally, the fluorescence was detected, and the relative ∆Ψm was analyzed by ImageJ software and is expressed as the red/green fluorescence pixel ratio. In addition, the ratio of polymers/monomer was detected by flow cytometry and analyzed by FlowJo V10 software.

### ATP detection

The ATP levels were detected via an ATP Assay Kit (Beyotime, S0027) according to the manufacturer’s instructions. Briefly, total protein of MSCs was extracted, and the concentrations were detected as described above. Then, 20 µl of protein solution was added to 100 µl of ATP working solution, and the luminescence intensity was detected with a luminometer. The ATP levels are expressed as nmol/mg of protein according to the standard curve.

### Mitochondrial ROS detection

A MitoSOX™ Red Mitochondrial Superoxide Indicator (Thermo Fisher, M36008) was used to detect the levels of mitochondrial ROS according to the manufacturer’s protocol. The MitoSOX probes were dissolved in DMSO and diluted with Hanks’ balanced salt solution (Thermo Fisher, 14025092). MSCs were stained with MitoSOX working solution (5 µM) for 15 min at 37 °C, and then, the nuclei were stained with DAPI. The MitoSOX signal was detected immediately and analyzed by ImageJ software.

### RNA stability detection

MSCs were seeded, and gene interference or overexpression was performed as described above. Seventy-two hours later, actinomycin D (2 µg/ml) was added to inhibit transcription, and total RNA was extracted at 0 min, 60 min, 120 min, 180 min and 240 min. The expression of target genes at different times was detected by qPCR, and the degradation rate was analyzed.

### Polysome profiling assay

Polysome profiling assays were performed by NKY-Genereader (Guangzhou, China). Briefly, MSCs transfected with ALKBH5-siRNA or NC-siRNA were treated with 100 µg/ml cycloheximide (MCE, HY-12320) for 10 min and collected. Then, the cytoplasm was extracted and layered onto a 10–50% sucrose gradient and centrifuged at 36,000 rpm for 2.5 h at 4 °C in an ultraspeed centrifuge (Beckman, L-100XP). The polysome fractions were collected, and the abundance of CYP1B1 mRNA was analyzed by qRT‒PCR.

### RNA pulldown

CYP1B1 mRNA and antisense transcript plasmids were purchased from Obio (Shanghai, China) and were transcribed in vitro by using a TranscriptAid T7 High-Yield Transcription Kit (Thermo Fisher, K0441). Then, a Pierce™ Magnetic RNA‒Protein Pull-Down Kit (Thermo Fisher, 20164) was used according to the manufacturer’s instructions. The transcripts were biotin-labeled and incubated with MSC lysates. Finally, the pulled-down proteins were obtained for western blot detection.

### RNA immunoprecipitation (RIP)

An EZ-Magna RIP™ RNA-Binding Protein Immunoprecipitation Kit (Millipore, 17–701) was used according to the manufacturer’s instructions. Briefly, MSCs were lysed and incubated with magnetic beads conjugated with anti-ALKBH5 (Proteintech, 16837–1-AP, 1:1,00), anti-IGF2BP1 (Abcam, ab184305, 1:1,00) or negative control IgG (Abcam, ab172730, 1:100). The immunoprecipitated RNAs were purified and extracted. The obtained RNAs and the input RNAs were subjected to qPCR followed by electrophoresis to detect the CYP1B1 mRNA level.

### Generation of conditional Alkbh5 knockout mice

The generation of conditional Alkbh5 knockout mice was performed as previously described^20^. Exon 1 of the Alkbh5 gene was selected as the conditional knockout region, and Alkbh5^fl/+^ mice with a C57BL/6 background were generated by Cyagen (Suzhou, China) using CRISPR/Cas-mediated genome engineering. Then, we crossed Prx1-Cre mice with Alkbh5^fl/+^ mice, and the genotypes of their offspring were identified. Offspring with the Prx1-Cre; Alkbh5^fl/+^ genotype were crossed with Alkbh5^fl/fl^ mice to obtain Prx1-Cre; Alkbh5^fl/fl^ mice, homozygous conditional Alkbh5 knockout mice. The genotype of the transgenic mice was identified by DNA electrophoresis of the PCR products that were amplified from genomic DNA extracted from mouse tails. The specific primers for Alkbh5 and Prx1-Cre are provided in Supplementary Table 1.

### OA model construction

An anterior cruciate ligament transection (ACLT)-induced OA model was constructed in mice. Briefly, 10-week-old male C57BL/6 mice were anesthetized, and the skin was prepared. The knee joint capsule was opened, and the ACL was carefully transected with microsurgical scissors under a microscope. For the sham operation, the right knee joints were exposed, but the ligament was not transected. At 1 week, 3 weeks, 5 weeks and 7 weeks after the operation, 10 µl of DMEM containing 1 * 10^5 control MSCs or ALKBH5-overexpressing MSCs was injected into the knee joint with an insulin syringe (BD Bioscience, 324702), with 10 µl of DMEM as a control. Eight weeks after the operation, the knee joints were collected for histological assessment.

### Tissue section preparation and histological staining

Mouse knee joints were collected and fixed in 10% neutral formalin for 1 day. Then, the joints were decalcified in rapid decalcification solution for 2 days and embedded in paraffin. Joint sections were prepared for further measurement.

For histological detection, the sections were deparaffinized and rehydrated. Then, they were stained with toluidine blue O (TBO), safranin O and fast green. The results were captured under a light microscope and quantified by using the Osteoarthritis Research Society International (OARSI) scoring system^21^.

### Immunofluorescence staining

The joint sections were deparaffinized and rehydrated, and antigen retrieval was performed by pepsin. The cells seeded on culture plates were fixed with 4% paraformaldehyde and treated with 1% Triton solution. Then, the sections or cells were blocked with 10% donkey serum for 0.5 h at room temperature and incubated with primary antibodies overnight at 4 °C. On the second day, they were washed and incubated with fluorophore-labeled secondary antibodies (Thermo Fisher, A48282 and A48287, 1:500) at room temperature for 1 h. Finally, an antifade solution containing 4′,6-diamidino-2-phenylindole (DAPI) was added, and the fluorescence signals were captured under a fluorescence microscope. The details of the primary antibodies are provided in the supplementary materials.

### Statistical analyses

The results of this study were analyzed by using SPSS 22.0 software. The data are expressed as the mean ± standard deviation (SD). Differences between different groups were analyzed by using independent-sample t tests (two groups) or one-way ANOVA (more than two groups). *P* < 0.05 was regarded as significant.

## Results

### The level of m6A modification increased and ALKBH5 was reduced during MSC senescence

**Fig. 1:**
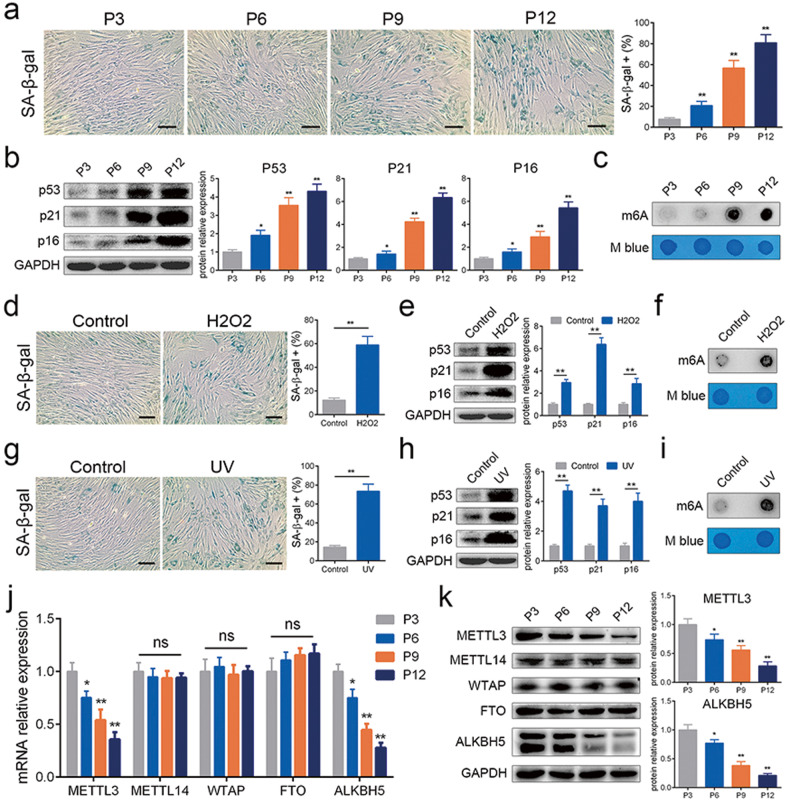
The level of m6A modification increased, and METTL3 and ALKBH5 levels were reduced during MSC senescence. **a** The percentages of SA-β-gal-positive cells increased with cell replication. **b** The protein levels of p53, p21 and p16 increased with cell replication. **c** The level of m6A modification increased with cell replication. **d** H_2_O_2_ treatment enhanced the percentages of SA-β-gal-positive cells. **e** H_2_O_2_ treatment enhanced the protein levels of p53, p21 and p16. **f** H_2_O_2_ treatment enhanced the level of m6A modification. **g** UV irradiation enhanced the percentages of SA-β-gal-positive cells. **h** UV irradiation enhanced the protein levels of p53, p21 and p16. **i** UV irradiation enhanced the level of m6A modification. **j** The mRNA levels of METTL3 and ALKBH5 decreased with cell replication. **k** The protein levels of METTL3 and ALKBH5 decreased with cell replication. *n* = 9, * indicates *P* < 0.05, ** indicates *P* < 0.01, ns indicates not significant, scale bar = 50 nm.

The characteristics of MSCs were tested and in accordance with international criteria (Supplementary Fig. 1)^19^. To determine whether m6A modification is involved in MSC senescence, we first induced MSC senescence with three classic methods: multiple replications, H_2_O_2_ stimulation and UV radiation. The proportion of SA-β-gal-positive cells was increased (Fig. 1a, d, g), and the expression of the cell cycle arrest-related proteins p53, p21 and p16 was enhanced (Fig. 1b, e, h) as the generation increased or with H_2_O_2_ or UV treatment, indicating the successful induction of MSC senescence. The results of the m6A dot blot analysis showed that the abundance of m6A modification was significantly increased in all three models (Fig. 1c, f, i). In addition, we detected the expression of crucial factors regarding m6A modification in different generations of MSCs. The results showed that the levels of METTL3 and ALKBH5 decreased as the cell generation increased (Fig. 1j, k), while the other genes did not change significantly (Fig. 1j, k and Supplementary Fig. 2a, b). Furthermore, we tested the expression of METTL3 and ALKBH5 in two other models of senescence and found that only the level of ALKBH5 decreased with H_2_O_2_ or UV treatment (Supplementary Fig. 2c–f). Together, these findings indicated that m6A may play a role in MSC senescence and that ALKBH5 may be the key factor.

### ALKBH5 deficiency facilitated MSC senescence

**Fig. 2:**
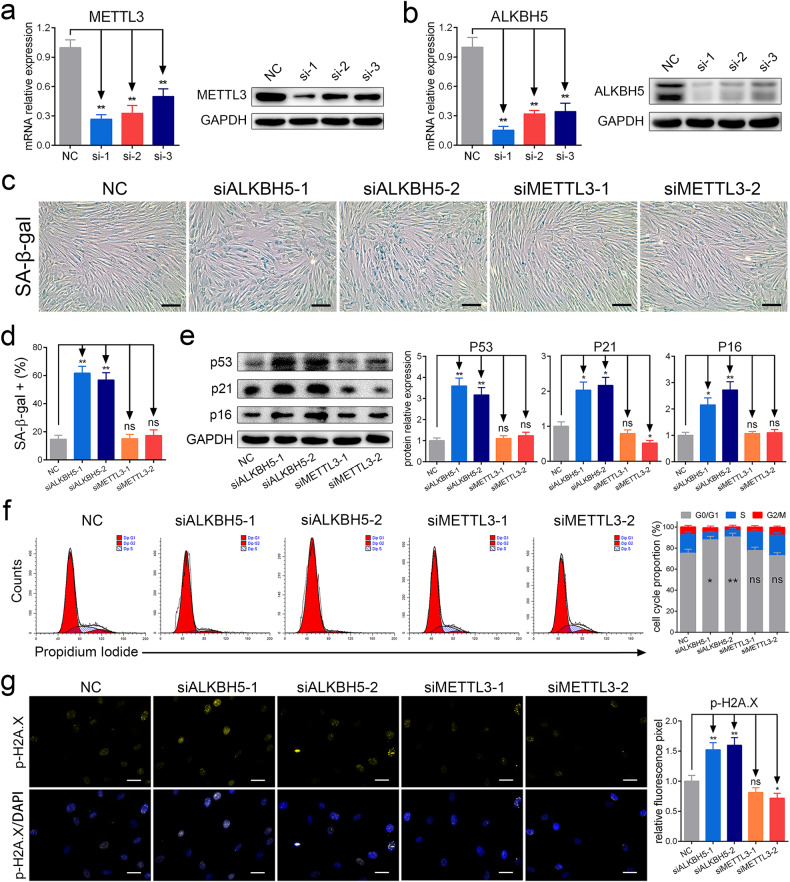
ALKBH5 deficiency facilitated MSC senescence. **a** The silencing efficiency of siRNAs targeting METTL3. **b** The silencing efficiency of siRNAs targeting ALKBH5. **c**, **d** Knockdown of ALKBH5 increased the percentages of SA-β-gal-positive cells, but knockdown of METTL3 had no significant effect (scale bar = 50 nm). **e** Knockdown of ALKBH5 increased the protein levels of p53, p21 and p16, and knockdown of METTL3 decreased the protein levels of p21 but had no significant effect on the protein levels of p53 or p16. **f** Knockdown of ALKBH5 increased the percentages of G0/G1 cells in the cell cycle, but knockdown of METTL3 had no significant effect. **g** Knockdown of ALKBH5 enhanced the p-H2A.X signal, but knockdown of METTL3 had no significant effect. Scale bar = 20 nm. *n* = 9, * indicates *P* < 0.05, ** indicates *P* < 0.01, ns indicates not significant.

To evaluate the effect of METTL3 and ALKBH5 on MSC senescence, we downregulated their expression in MSCs by siRNAs. The interference efficiency of the siRNAs is shown (Fig. 2a, b), and two of the more efficient siRNAs were selected for further experiments. The results showed that both siRNAs targeting ALKBH5 increased the number of SA-β-gal-positive cells in MSCs, while siRNAs targeting METTL3 had no significant effect (Fig. 2c, d). The β-gal enzymatic assay showed similar results (Supplementary Fig. 3a). In addition, the levels of p53, p21 and p16 were significantly elevated with downregulation of ALKBH5 expression but not METTL3 expression (Fig. 2e). We also assessed the cell cycle of MSCs and found that interfering with ALKBH5 helped block MSCs in G0/G1 phase, while interfering with METTL3 resulted in no significant changes (Fig. 2f). Furthermore, we detected the fluorescence signal of p-H2A.X^22^, a marker of DNA damage that often occurs with cellular senescence, and the results showed that knockdown of ALKBH5 strengthened the p-H2A.X signal (Fig. 2g). Moreover, similar results were found for the mean fluorescence intensity (MFI) of p-H2A.X detected by flow cytometry (Supplementary Fig. 4). Given that the effect of siRNA is transient, we constructed lentiviruses for stable knockdown of ALKBH5 or METTL3, and we found that stable knockdown of ALKBH5 facilitated MSC senescence, while stable knockdown of METTL3 had no significant effect (Supplementary Fig. 5a–f). We also detected the level of m6A modification, and the results showed that both siRNAs targeting ALKBH5 enhanced the level of m6A modification in MSCs (Supplementary Fig. 6a). Together, these results revealed that ALKBH5 deficiency facilitated MSC senescence.

### ALKBH5 alleviated MSC senescence via m6A demethylation activity

**Fig. 3:**
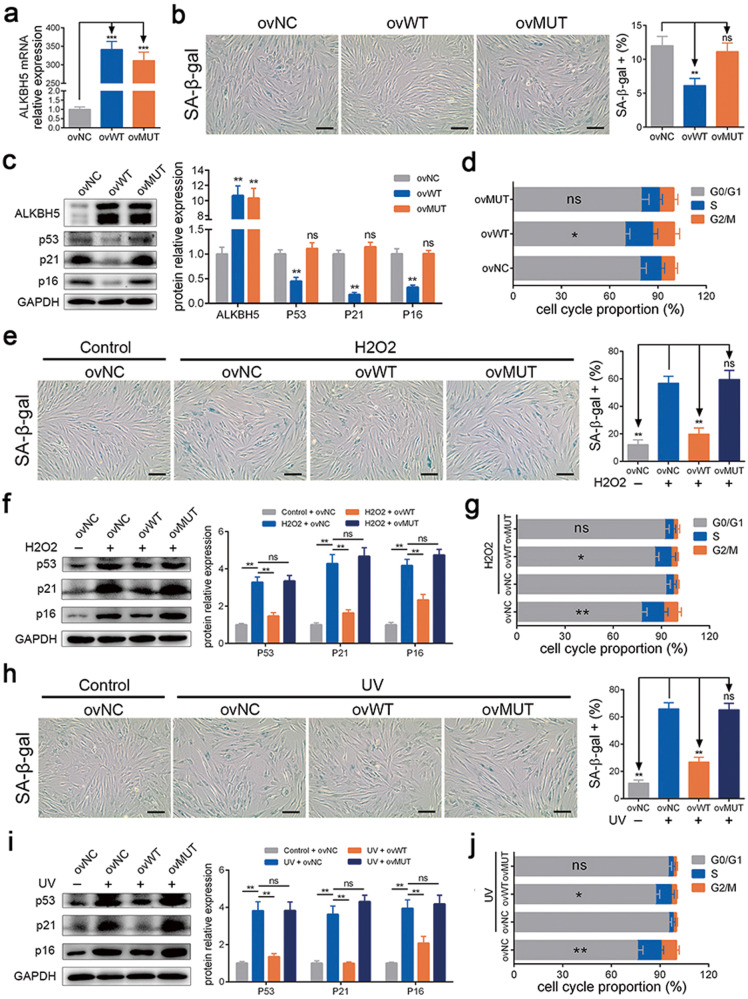
ALKBH5 alleviated MSC senescence via m6A demethylation activity. **a** The overexpression efficiency of WT-ALKBH5 and MUT-ALKBH5. **b** WT-ALKBH5 reduced the percentages of SA-β-gal-positive cells, but MUT-ALKBH5 had no significant effect. **c** WT-ALKBH5 reduced the protein levels of p53, p21 and p16, but MUT-ALKBH5 had no significant effect. **d** WT-ALKBH5 reduced the percentages of G0/G1 in the cell cycle, but MUT-ALKBH5 had no significant effect. **e** WT-ALKBH5 improved the effect of H_2_O_2_ on SA-β-gal staining, but MUT-ALKBH5 had no significant effect. **f** WT-ALKBH5 improved the effect of H_2_O_2_ on the protein levels of p53, p21 and p16, but MUT-ALKBH5 had no significant effect. **g** WT-ALKBH5 improved the effect of H_2_O_2_ on the percentages of G0/G1 in the cell cycle, but MUT-ALKBH5 had no significant effect. **h** WT-ALKBH5 improved the effect of UV on SA-β-gal staining, but MUT-ALKBH5 had no significant effect. **i** WT-ALKBH5 improved the effect of UV on the protein levels of p53, p21 and p16, but MUT-ALKBH5 had a nonsignificant effect. **j** WT-ALKBH5 improved the effect of UV on the percentages of G0/G1 in the cell cycle, but MUT-ALKBH5 had no significant effect. *n* = 9, * indicates *P* < 0.05, ** indicates *P* < 0.01, ns indicates not significant, scale bar = 50 nm.

To further determine the function of ALKBH5 in MSC senescence, we upregulated ALKBH5 expression by using an overexpression lentivirus (Fig. 3a, c). Overexpression of WT ALKBH5 reduced the strength of SA-β-gal staining, β-gal activity, the expression of p53, p21, and p16 and the percentage of G0/G1 phase cells in MSCs (Fig. 3b–d and Supplementary Fig. 3b). We also overexpressed a mutant (MUT), ALKBH5 (H204A), which lacks m6A demethylation activity, and the results showed that mutant ALKBH5 had no significant effect. In addition, we found that WT ALKBH5 but not MUT ALKBH5 helped alleviate MSC senescence induced by H_2_O_2_ stimulation (Fig. 3e–g and Supplementary Fig. 3c) or UV radiation (Fig. 3h–j and Supplementary Fig. 3d). Furthermore, ALKBH5 overexpression reduced the levels of m6A modification induced by multiple replications, H_2_O_2_ stimulation or UV irradiation (Supplementary Fig. 6b–d). Together, these results indicated that ALKBH5 alleviated MSC senescence through its m6A demethylation activity.

### CYP1B1 is the downstream target of ALKBH5 in the regulation of senescence

**Fig. 4:**
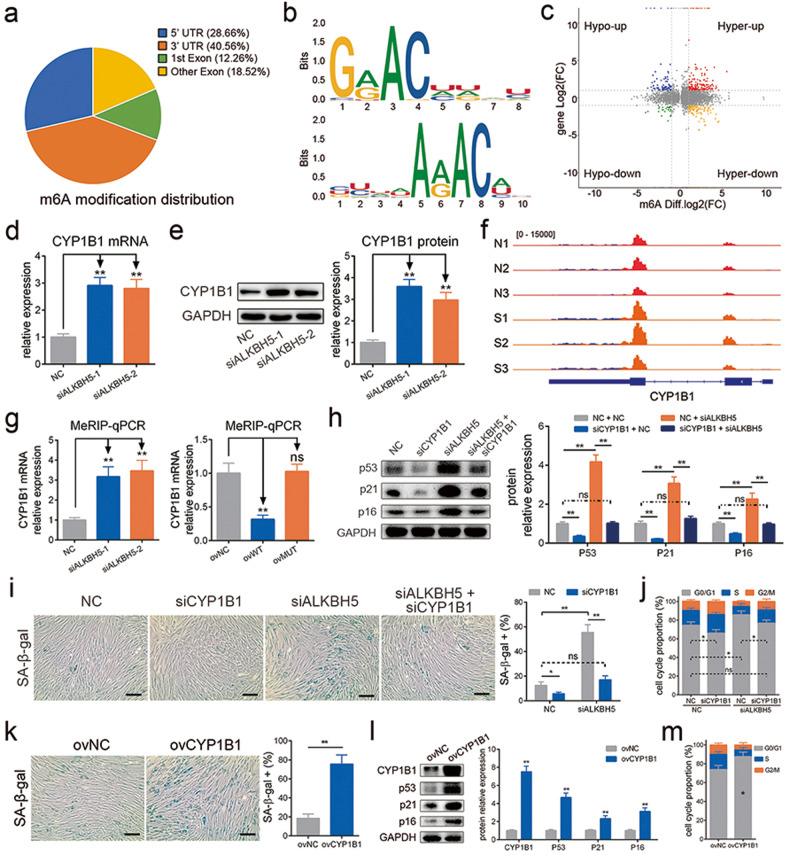
CYP1B1 is the downstream target of ALKBH5 in senescence regulation. **a** The m6A modification distribution of the MeRIP-Seq data. **b** The consensus sequence motifs for m6A methylation in the MeRIP-Seq. **c** The conjoint analysis of m6A modification and gene expression. **d** Knockdown of ALKBH5 increased the mRNA level of CYP1B1. **e** Knockdown of ALKBH5 increased the protein level of CYP1B1. **f** The m6A peaks diagram showed that the m6A abundance on CYP1B1 mRNA increased in the ALKBH5-silencing group. **g** MeRIP-qPCR showed that knockdown of ALKBH5 increased and overexpression of WT-ALKBH5 reduced the level of m6A modification on CYP1B1 mRNA but MUT-ALKBH5 had no significant effect. **h** Knockdown of CYP1B1 reduced the protein levels of p53, p21 and p16 and rescued the effect of ALKBH5 knockdown on them. **i** Knockdown of CYP1B1 reduced the percentages of SA-β-gal-positive cells and rescued the effect of ALKBH5 knockdown. **j** Knockdown of CYP1B1 reduced the percentages of G0/G1 cells in the cell cycle and rescued the effect of ALKBH5 knockdown. **k** Overexpression of CYP1B1 increased the percentage of SA-β-gal-positive cells. **l** Overexpression of CYP1B1 increased the protein levels of p53, p21 and p16. **m** Overexpression of CYP1B1 increased the percentage of G0/G1 cells in the cell cycle. *n* = 9, * indicates *P* < 0.05, ** indicates *P* < 0.01, ns indicates not significant, scale bar = 50 nm.

To further explore the mechanism of ALKBH5 in MSC senescence, we performed MeRIP-Seq and RNA-Seq to detect the m6A modification profile and gene expression profile in MSCs with ALKBH5 knockdown. The results showed that the preferential location of m6A on transcripts in MSCs was the 3′ untranslated region (UTR) (40.56%) and the 5′ UTR (28.66%) (Fig. 4a). The distance profile showed that m6A peak calls were enriched around the transcriptional start site (TSS) and transcriptional end site (TES) (Supplementary Fig. 7a). The consensus sequence motifs for m6A methylation identified in our sequences were consistent with the m6A motif “RRACH” (Fig. 4b). The conjoint analysis of m6A modification and gene expression was performed (Fig. 4c), and the heatmap of differentially expressed genes is shown (Supplementary Fig. 7b). Peak calls with a fold change ≥ 2 and *P* value < 0.05 were regarded as significantly differentially expressed, and the m6A modification of 841 genes was strengthened and that of 209 genes was weakened with ALKBH5 knockdown. More genes acquired stronger m6A modification with ALKBH5 knockdown, which was consistent with the downregulation of demethylase expression. In addition, Gene Ontology (GO) enrichment analyses and Kyoto Encyclopedia of Genes and Genomes (KEGG) pathway analyses showed that the differentially expressed genes were enriched in the “Cell Cycle” term and were related to the “Cell Cycle” and “Cellular Senescence” pathways (Supplementary Fig. 7c, d), which was consistent with our finding that ALKBH5 deficiency induced cell cycle arrest and senescence in MSCs.

From the MeRIP-Seq data, we screened several genes, including CDK6, CCNE1, CYP1B1, PSMC1 and PSMF1, which acquired stronger m6A modification with ALKBH5 knockdown and were involved in the cell cycle or cellular senescence. We measured the expression of these genes and found that knockdown of ALKBH5 elevated both the mRNA and protein levels of CYP1B1 (Fig. 4d, e) but had no obvious effect on the other genes (Supplementary Fig. 8a, b). In addition, the m6A peaks of CYP1B1 from the MeRIP-Seq data are shown (Fig. 4f), and MeRIP-qPCR analysis verified that interfering with ALKBH5 strengthened the m6A modification of CYP1B1 (Fig. 4g). In addition, WT ALKBH5 but not MUT ALKBH5 enhanced both the m6A modification level (Fig. 4g) and the expression of CYP1B1 (Supplementary Fig. 8c, d). Furthermore, the results of the RIP assay and RNA pulldown revealed an interaction between the ALKBH5 protein and CYP1B1 mRNA (Supplementary Fig. 8e, f). These data revealed that CYP1B1 served as a downstream target of ALKBH5 via m6A modification.

We further determined the effect of CYP1B1 on MSC senescence. First, we found that the expression of CYP1B1 in MSCs increased during cellular senescence induced by multiple replications, H_2_O_2_ or UV (Supplementary Fig. 9a–c). Next, we downregulated the expression of CYP1B1 by siRNAs, and the siRNA with the highest efficiency was chosen for the following experiment (Supplementary Fig. 9d). CYP1B1 knockdown lowered the expression of p53, p21, and p16, the percentage of SA-β-gal-positive cells, the activity of β-gal and the proportion of G0/G1 phase cells in MSCs and reversed the effect of downregulation of ALKBH5 expression on MSC senescence (Fig. 4h–j and Supplementary Fig. 3e). In addition, we overexpressed CYP1B1 by lentivirus and found that CYP1B1 dramatically facilitated MSC senescence, as indicated by the increased level of SA-β-gal staining, β-gal activity, cell cycle arrest proteins and G0/G1 phase ratio (Fig. 4k–m and Supplementary Fig. 3f). These findings indicated that CYP1B1 is the downstream gene of ALKBH5 responsible for the regulation of MSC senescence.

### ALKBH5 regulated MSC senescence though CYP1B1 via mitochondrial dysfunction

**Fig. 5:**
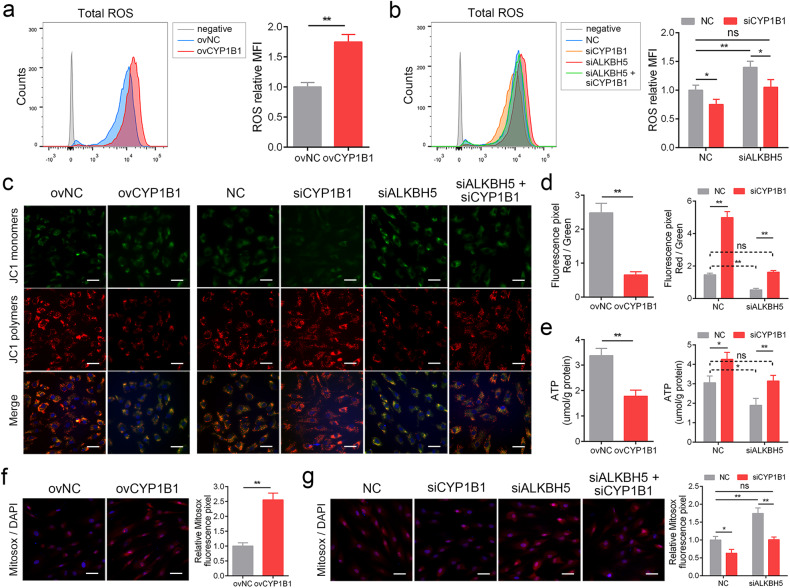
ALKBH5 regulated MSC senescence though CYP1B1 via mitochondrial dysfunction. **a** Overexpression of CYP1B1 increased the level of ROS. **b** Knockdown of CYP1B1 reduced the level of ROS and rescued the effect of ALKBH5 knockdown. **c**, **d** Overexpression of CYP1B1 impaired the MMP, and knockdown of CYP1B1 elevated the MMP and rescued the effect of ALKBH5 knockdown, scale bar = 25 nm. **e** Overexpression of CYP1B1 impaired the level of ATP, and knockdown of CYP1B1 elevated the level of ATP and rescued the effect of ALKBH5 knockdown. **f** Overexpression of CYP1B1 impaired the signal of MitoSOX, scale bar = 50 nm. **g** Knockdown of CYP1B1 elevated the signal of MitoSOX and rescued the effect of ALKBH5 knockdown. Scale bar = 50 nm. *n* = 9, * indicates *P* < 0.05, ** indicates *P* < 0.01, ns indicates not significant.

Then, we investigated how CYP1B1 led to MSC senescence. CYP1B1 was shown to be mainly located in mitochondria and might be involved in mitochondrial dysfunction and ROS production, two crucial causes of cellular senescence^17,23^. Therefore, we detected the effect of CYP1B1 on mitochondrial function and ROS production in MSCs. The results showed that overexpression of CYP1B1 significantly enhanced the level of total ROS in MSCs (Fig. 5a). Likewise, silencing ALKBH5 also promoted total ROS accumulation in MSCs, and this effect was successfully abrogated by CYP1B1 knockdown (Fig. 5b). Subsequently, we evaluated the function of mitochondria by detecting mitochondrial membrane potential (MMP), ATP production and mitochondrial ROS. The results showed that upregulation of CYP1B1 expression impaired MMP (Fig. 5c, d and Supplementary Fig. 10a) and ATP production (Fig. 5e) but increased mitochondrial ROS signaling (Fig. 5f) in MSCs. In addition, knockdown of ALKBH5 led to similar results, and silencing CYP1B1 rescued these effects (Fig. 5c–e, g and Supplementary Fig. 10b). Furthermore, we overexpressed CYP1B1 in MSCs by adding N-acetylcysteine (NAC) or beta-mercaptoethanol (β-ME), two reducing agents able to eliminate ROS. The results showed that both NAC and β-ME could reverse the effect of CYP1B1 on the percentage of SA-β-gal-positive cells, β-gal activity, the protein levels of p53, p21, and p16 and the ratio of G0/G1 phase in MSCs (Supplementary Fig. 11a–d). Together, these results indicated that ALKBH5 and CYP1B1 modulated MSC senescence by regulating mitochondrial dysfunction and ROS production.

### Knockdown of ALKBH5 enhanced CYP1B1 mRNA stability in an IGF2BP1-dependent manner

**Fig. 6:**
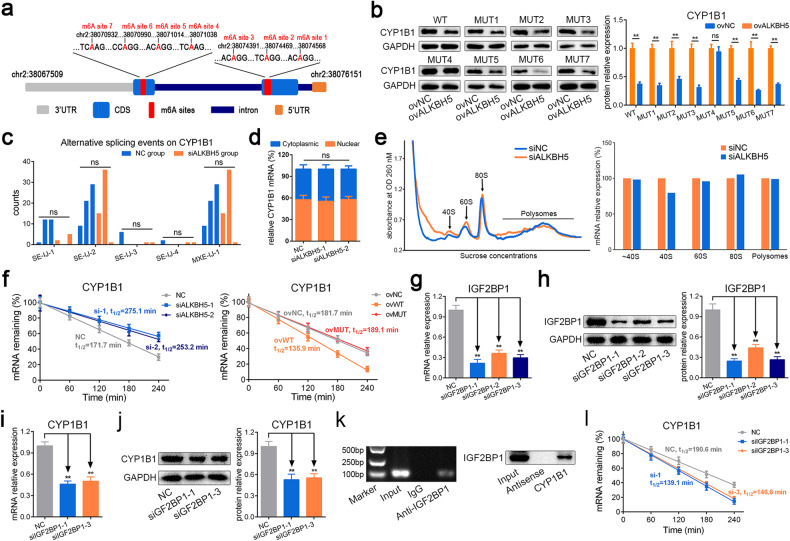
Knockdown of ALKBH5 enhanced CYP1B1 mRNA stability in an IGF2BP1-dependent manner. **a** The m6A sites predicted with high confidence on CYP1B1 mRNA. **b** Overexpression of ALKBH5 reduced the protein levels of CYP1B1 in the WT-CYP1B1 group and other MUT groups except for the MUT4 group. **c** The alternative splicing events of CYP1B1 analyzed from the RNA-Seq data by rMATS. **d** The nuclear and cytoplasmic distribution of CYP1B1 mRNA was not changed significantly with knockdown of ALKBH5. **e** The abundance of CYP1B1 mRNA on polysomes was not changed significantly with knockdown of ALKBH5. **f** Knockdown of ALKBH5 reduced the degradation rate of CYP1B1 mRNA, and WT-ALKBH5 facilitated the degradation rate of CYP1B1 mRNA but MUT-ALKBH5 had no significant effect. **g** The silencing efficiency of siRNAs targeting IGF2BP1 at the mRNA level. **h** The silencing efficiency of siRNAs targeting IGF2BP1 at the protein level. **i** Knockdown of IGF2BP1 reduced the mRNA level of CYP1B1. **j** Knockdown of IGF2BP1 reduced the protein level of CYP1B1. **k** RIP assays showed that CYP1B1 mRNA was precipitated by IGF2BP1 antibody (left), and RNA pulldown assays showed that IGF2BP1 protein was pulled down by CYP1B1 mRNA (right). **l** Knockdown of IGF2BP1 facilitated the degradation of CYP1B1 mRNA. *n* = 9, ** indicates *P* < 0.01, ns indicates not significant.

We further explored how ALKBH5 mediates the expression of CYP1B1 via m6A modification. First, we predicted the m6A sites of CYP1B1 mRNA on the m6A-Atlas, m6Avar and SMARP websites^24–26^. The results showed dozens of predicted sites, and most of them were located in the coding DNA sequence (CDS) of CYP1B1 mRNA, which is consistent with the m6A peak diagram of CYP1B1 from our MeRIP-Seq data (Fig. 4g). We selected 7 sites with high confidence and constructed plasmids overexpressing the CYP1B1 CDS with the corresponding point mutations (Fig. 6a). Then, we silenced ALKBH5 and transfected WT or MUT CYP1B1 plasmids into MSCs and found that the expression of CYP1B1 was not changed significantly in the MUT4 group (Fig. 6b), indicating that m6A site 4 on CYP1B1 mRNA was the critical site mediated by ALKBH5.

Next, we explored the effect of ALKBH5-regulated m6A modification on CYP1B1 mRNA. Previous reports have shown that m6A modification usually regulates the stability, translation efficiency, cellular translocation or splicing of mRNA^11^. We analyzed the alternative splicing events of CYP1B1 from the RNA-Seq data by rMATS, and the results showed that skipped exons (SEs) and mutually exclusive exons (MXEs) occurred in CYP1B1. However, the counts of SE events and MXE events were not significantly changed in the ALKBH5-silenced group (Fig. 6c). In addition, we measured the nuclear and cytoplasmic proportions of CYP1B1 mRNA with ALKBH5 knockdown, but we obtained negative results (Fig. 6d). Then, to explore whether ALKBH5 regulates the translation efficiency of CYP1B1, we performed a polysome profiling assay, and the results showed that the abundance of CYP1B1 mRNA on polysomes was not changed with ALKBH5 knockdown (Fig. 6e). Furthermore, we detected the degradation rate of CYP1B1 mRNA and found that knockdown of ALKBH5 notably enhanced the stability of CYP1B1 mRNA (Fig. 6f). In addition, upregulation of WT ALKBH5 expression accelerated the degradation of CYP1B1 mRNA, but MUT ALKBH5 had no significant effect (Fig. 6f). These findings indicated that ALKBH5 reduced the expression of CYP1B1 by impairing the stability of CYP1B1 mRNA.

We further explored the m6A “readers” regulating CYP1B1. Among the known “readers” of m6A modification, IGF2BP1/2/3 have been reported to enhance the stability of transcripts^27^. Therefore, we downregulated IGF2BP1/2/3 expression in MSCs and found that knockdown of IGF2BP1 but not IGF2BP2 or IGF2BP3 increased CYP1B1 at both the mRNA and protein levels (Fig. 6g–j and Supplementary Fig. 12a–h). In addition, the results of RNA pulldown on CYP1B1 mRNA revealed an interaction between IGF2BP1 and CYP1B1 mRNA, and the RIP assays of IGF2BP1 obtained a similar outcome (Fig. 6k). Furthermore, we found that knockdown of IGF2BP1 accelerated the degradation rate of CYP1B1 mRNA (Fig. 6l). Together, our data indicated that IGF2BP1 was the m6A “reader” on CYP1B1 mRNA, which helped improve its stability.

### Conditional Alkbh5 knockout mice displayed severe OA, and Alkbh5 improved the efficacy of MSCs in ACLT-induced OA

**Fig. 7:**
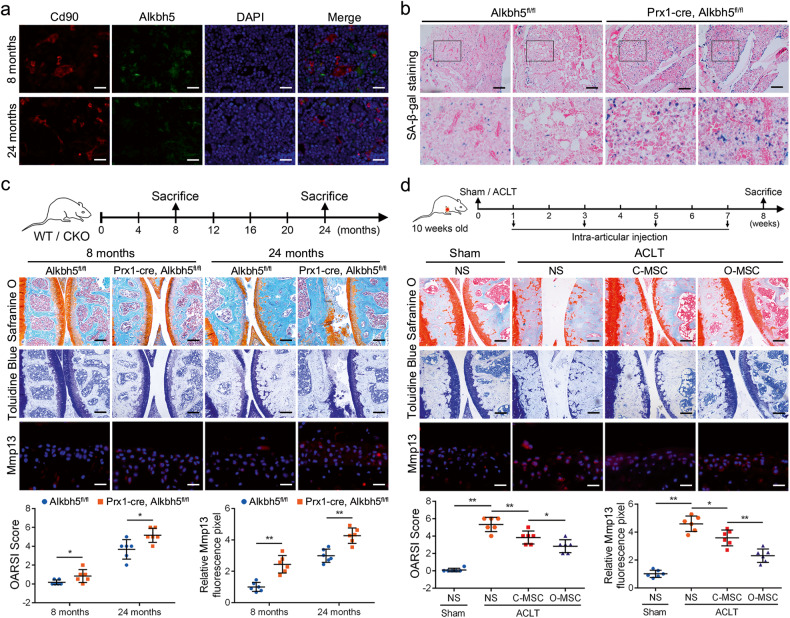
Conditional Alkbh5 knockout mice displayed a severe OA phenotype, and Alkbh5 improved the efficacy of MSCs in ACLT-induced OA. **a** The Alkbh5 signal on Cd105-positive cells was impaired in 24-month-old mice compared to 8-month-old mice; scale bar = 40 nm. **b** SA-β-gal staining was stronger in the Prx1-cre; Alkbh5^fl/fl^ mice than in the Alkbh5^fl/fl^ control littermates; scale bar = 200 nm. **c** Both at 8 months and 24 months, the OARSI score and Mmp13 signal of the knee joint were increased in the Prx1-cre; Alkbh5^fl/fl^ mice compared to the Alkbh5^fl/fl^ mice. **d** The knee joints of the ACLT group mice had more severe OARSI scores and higher Mmp13 expression. Intra-articular injection of control MSCs (C-MSCs) improved the OARSI score and Mmp13 expression, while overexpressing ALKBH5 MSCs (O-MSCs) had a better effect; scale bar = 200 nm (top and middle), scale bar = 40 nm (bottom). *n* = 6, * indicates *P* < 0.05, ** indicates *P* < 0.01, ns indicates not significant.

OA is a common age-related disease, and MSCs play an important role in the development and treatment of OA^8,28,29^. To further evaluate the role of ALKBH5 and CYP1B1 in aging and OA, we constructed an aging mouse model and transgenic mice with Alkbh5 knockout specifically in MSCs, Prx1-Cre; Alkbh5^fl/fl^ mice (Supplementary Fig. 13). We first collected knee joints of mice and measured the expression of Alkbh5 in bone marrow. The immunofluorescence results showed that compared to that in the young mice, the signal of Alkbh5 in MSCs was reduced in the aged mice (Fig. 7a and Supplementary Fig. 14a). The western blot results also revealed that the levels of Alkbh5 were reduced and the levels of Cyp1b1 were elevated in the aged mice and the Prx1-Cre; Alkbh5^fl/fl^ mice (Supplementary Fig. 14b). In addition, the m6A modification levels were enhanced in the aged mice and Prx1-Cre; Alkbh5^fl/fl^ mice (Supplementary Fig. 14c). Furthermore, SA-β-gal staining showed more senescent cells in the subfacial bone marrow of the knee joint of the Prx1-Cre; Alkbh5^fl/fl^ mice than in that of the Alkbh5^fl/fl^ control littermates (Fig. 7b). To assess the degree of OA, we performed safranin O and TBO staining, and the results showed that cartilage defects and the OARSI score were more severe in the Prx1-Cre; Alkbh5^fl/fl^ mice (Fig. 7c). In addition, the expression of Mmp13, a key matrix metalloproteinase in the development of OA, was enhanced in the Prx1-Cre; Alkbh5^fl/fl^ mice (Fig. 7c and Supplementary Fig. 14d, e). Furthermore, we evaluated the effect of Alkbh5 on the therapeutic effect of MSCs in the mice with ACLT-induced OA. The results showed that intra-articular injection of control MSCs helped alleviate cartilage defects, OARSI scores and Mmp13 expression (Fig. 7d and Supplementary Fig. 14f, g). Importantly, compared to the control MSCs, the Alkbh5-overexpressing MSCs displayed better efficacy (Fig. 7d and Supplementary Fig. 14f, g). Together, these findings indicated that knockout of Alkbh5 in MSCs facilitated OA development and that overexpression of Alklbh5 enhanced the efficacy of MSCs in OA treatment.

## Discussion

In this study, we focused on the function of m6A modification in MSC senescence and the age-related disease OA. Our data showed that the m6A level was enhanced and that the demethylase ALKBH5 had downregulated expression during MSC senescence. Mechanistically, knockdown of ALKBH5 increased the stability of CYP1B1 mRNA in an IGF2BP1-dependent manner, which further caused mitochondrial dysfunction and resulted in senescence of MSCs. In addition, specific knockout of Alkbh5 in MSCs intensified OA in mice, and Alkbh5 helped improve the efficacy of MSC infusion in OA.

Longevity or prolonging the period of health is our eternal pursuit, and cellular senescence is one of the most critical factors^1^. Cell senescence is associated with many kinds of organ dysfunction and disease development, especially that of stem cells^1^. Previously, researchers have explored the mechanism and function of stem cell senescence and revealed its promising application in improving health and prolonging life. For example, Xiao et al.^30^ showed that hypothalamic stem cell senescence was involved in physiological decline and could be pharmacologically targeted to improve aging-related outcomes. Zhang et al.^31^ found that inhibiting muscle stem cell senescence helped improve cell dysfunction and extend lifespan in mammals. Here, we explored the senescence of MSCs and revealed that knockout of ALKBH5 in MSCs facilitated cellular senescence and aggravated OA in mice, which further indicated the correlation between stem cell senescence and senile disorders and provided new insights into the prevention and treatment of age-related diseases.

m6A is the most abundant type of RNA modification and has attracted much attention from scholars in the last few years^10^. Previous studies have shown that m6A participates in the fate and functional regulation of stem cells. Bertero et al.^32^ showed that METTL3-METTL14-WTAP complex-mediated m6A modification is involved in the early cell fate decisions of human pluripotent stem cells. Wu et al.^33^ revealed the pathological outcomes of m6A dysregulation in MSC differentiation and osteoporosis. However, little is known about the role of m6A modification in stem cell senescence. In our study, we explored the role of m6A in MSC senescence and found that the level of m6A modification was enhanced and the expression of METTL3 and ALKBH5 was decreased during MSC aging. In addition, ALKBH5 protected against cellular senescence via its m6A demethylation activity. Recently, several studies have revealed the role of METTL3 and m6A in cellular senescence and consistently found that the level of METTL3 was changed in aging cells and that m6A modification played a role in regulating cellular senescence^34–36^. For instance, Wu’s study reported that both m6A modification and METTL3 were reduced in prematurely senescent MSCs and that METTL3 alleviated MSC senescence through m6A modification-dependent function^34^. However, this study did not determine the expression of other m6A-related genes in senescent MSCs. In addition, the changes in the m6A level and the function of METTL3 in MSC senescence in this study were not consistent with our previous results. In that study, two premature aging cellular models created by gene mutation were used, while our study used three senescence cellular models induced by replication, H_2_O_2_ stimulation or UV irradiation. The difference in models may be the cause of the different results, which suggests that there are diverse mechanisms and functions of m6A modification in different aging conditions. In addition, we are the first to comprehensively explore the role of m6A-related genes in three classic models of cell senescence. Moreover, these three models are closely related to human aging and diseases; thus, our findings about the function of m6A and ALKBH5 in senescence have physiological and pathological importance.

The effect of m6A modification on RNA requires the participation of m6A readers. Different m6A readers have different effects on RNA regulation^37^. YTHDC1 promotes exon inclusion by recruiting splicing factors or facilitates the nuclear export of m6A-modified mRNAs by interacting with RNA export factors^37^. IGF2BPs inhibit RNA degradation by recruiting mRNA stabilizers such as HuR and facilitate translation by promoting ribosome loading and interactions with initiation factors^27^. In our study, we detected the splicing, cellular distribution, translation efficiency and degradation rate of CYP1B1 mRNA and found that only the degradation rate was reduced in the m6A-modified CYP1B1 transcript following ALKBH5 knockdown. In addition, we found that IGF2BP1 binds to CYP1B1 mRNA and inhibits its degradation, which is consistent with previous reports showing that IGF2BPs identify m6A modifications and enhance the stability of transcripts. In addition, our data showed that although the level of IGF2BP1 was not changed during MSC senescence, this molecule participated in the aging process by regulating the expression of CYP1B1 as the abundance of m6A changed. Similarly, previous studies also indicated a role of IGF2BPs in cell growth and aging. Ramanan et al.^38^ identified the novel potential influence of IGF2BP3 on tau pathology, which contributes to Alzheimer’s disease. Wu et al.^34^ identified IGF2BP2 as a key player in stabilizing m6A-modified MIS12 mRNA and attenuating premature aging. Together, IGF2BPs may be promising targets for aging prevention and should be further studied.

Cytochrome P450 is an important kind of metabolic enzyme that is mainly located in the mitochondria and is involved in degenerative diseases. For instance, cytochrome P450 metabolites of polyunsaturated fatty acids have a positive role in neurodegenerative diseases^39^. CYP2J2 was shown to attenuate age-related insulin resistance and metabolic dysfunction^40^. However, little is known about the role of cytochrome P450 enzymes in cellular senescence. Here, we first reported that an important cytochrome P450 monooxygenase, CYP1B1, facilitates MSC senescence. In addition, we revealed that CYP1B1 induced mitochondrial dysfunction and excess ROS production, two major causes of senescence, in MSCs. Previous studies have indicated that CYP1B1 might be responsible for excess ROS generation and that inhibition of CYP1B1 could attenuate mitochondrial dysfunction^17,41^, which is consistent with our findings. However, the mechanism underlying CYP1B1-induced mitochondrial dysfunction is still unclear. The electron transfer chain (ETC) is vital for mitochondrial metabolism and ROS production^42^. Previous studies have shown that cytochrome P450 plays a critical role in electron transfer and revealed some mechanisms. The CYP116 family contains an N-terminal heme domain fused to a phthalate dioxygenase-type reductase domain and acquires electrons from the NA(D)PH-FMN pathway^43^. CP2E1 contributed to the prevention of H_2_O_2_-induced inactivation during electron transfer^44^. We believe that CYP1B1 may mediate mitochondrial dysfunction and superoxide production by impacting the ETC, and further research is required to assess this possibility in the future.

OA is a common degenerative disease affecting millions of people, with chronic inflammation and cartilaginous injury as the main pathological features^45^. Recent studies revealed that aberrant MSCs in subchondral bone could trigger OA onset and that knockout of Tgfbr2 in MSCs attenuated the development of OA in mice^29^. In addition, clearance of senescent cells helped improve OA, and joint injections of aged MSCs caused OA-like cartilage degradation^8^. In our study, we observed that the expression of Alkbh5 was decreased in subchondral MSCs of the knee joint acquired from aged mice. In addition, knockout of Alkbh5 in MSCs accelerated cellular senescence of bone marrow and aggravated the pathogenesis of OA in mice. Previous reports and our findings jointly indicated that MSC senescence is a critical pathogenic factor of OA. Then, how do senescent MSCs lead to the development of OA? We suspect that, first, MSCs exist in the capsule, synovium and subchondral bone of joints, and when they become senescent, MSCs develop SASPs and secrete large amounts of cytokines, including IL-6, MCP-1, TGF-β and matrix metalloproteinase, which could cause inflammation and cartilage defects in OA^46^. Second, MSCs are an important source of chondroblasts and are crucial for tissue repair^6^. The capacities of cartilage differentiation and repair in MSCs are impaired following senescence^7^. Third, senescent MSCs may cause senescence of adjacent cells in the articular microenvironment, such as chondrocytes and fibroblasts, jointly leading to articular aging and degeneration^47^. Studies to further illustrate the mechanism of MSC senescence in the pathogenesis of OA are needed in the future.

MSC infusion is widely used in various disorders, and several clinical trials have shown that intra-articular injection of MSCs achieved pain relief and functional improvement in OA patients^48,49^. Our study also showed that MSC infusion is effective for OA. However, there are still many challenges for stem cell therapy, and further improvement in efficacy is needed^50^. Many studies have aimed to enhance the curative effect of MSCs by modifying their properties and have provided some potential regulatory targets. In terms of OA, the inflammatory factors and oxidative stress of the joint microenvironment may adversely affect the status of MSCs, such as inducing senescence, and impact their efficacy. Here, we first found that overexpression of ALKBH5 in MSCs could improve their efficacy in OA treatment, which may be attributed to the ability of ALKBH5 to protect against stress and alleviate cellular senescence. Our data suggest that antisenescence methods are a promising strategy to improve the effectiveness of MSC therapy and that ALKBH5 is a potential target. More preclinical and clinical trials are required for further investigation and applications.

In conclusion, our study first revealed that ALKBH5 regulated MSC senescence through m6A modification of CYP1B1 and provided promising targets for the treatment of age-related disorders and improvement in the efficacy of MSCs.

## Supplementary information

Supporting Information

## Data Availability

The data of this study are available from the corresponding authors on reasonable request.
