# Supplementary material for: ALKBH5 facilitates CYP1B1 mRNA degradation via m6A demethylation to alleviate MSC senescence and osteoarthritis progression
Source: Exp Mol Med. 2023 Aug 1;55(8):1743–56. doi: 10.1038/s12276-023-01059-0 (PMC10474288; doi:10.1038/s12276-023-01059-0)
Supplement: Supplementary file 1 — Supporting Information [file 12276_2023_1059_MOESM1_ESM.pdf]

Supplementary Figures

Supplementary Fig. 1

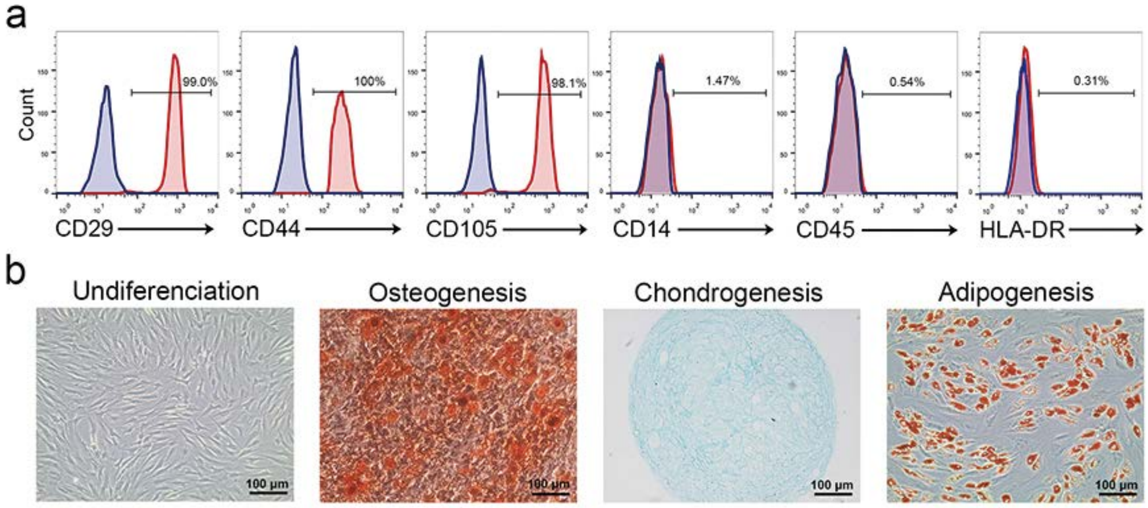

Supplementary Fig. 2

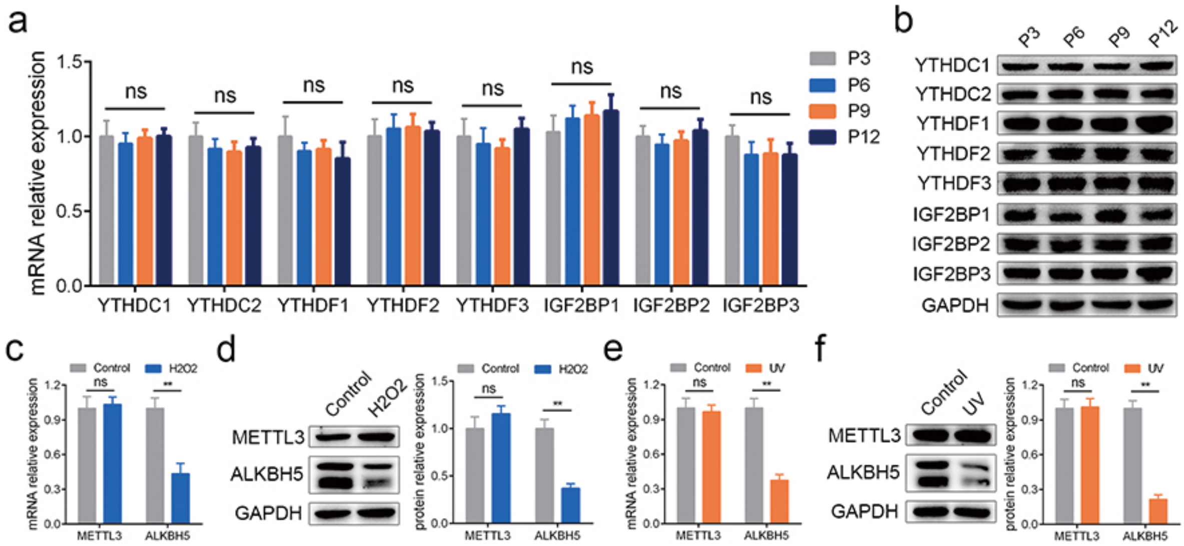

Supplementary Fig. 3

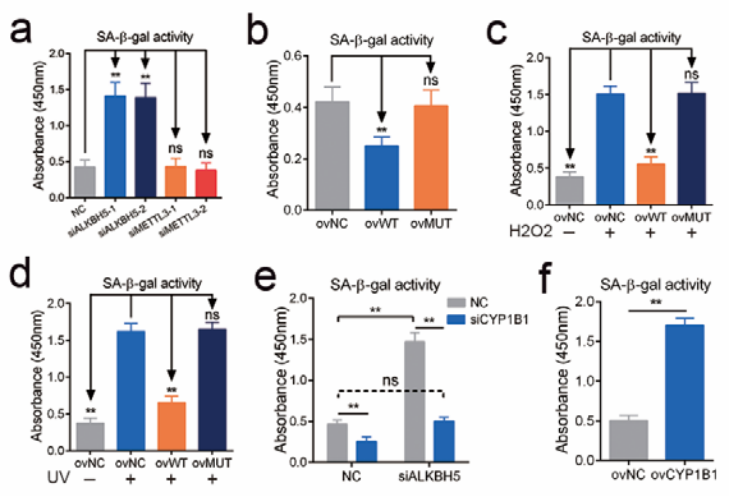

Supplementary Fig. 4

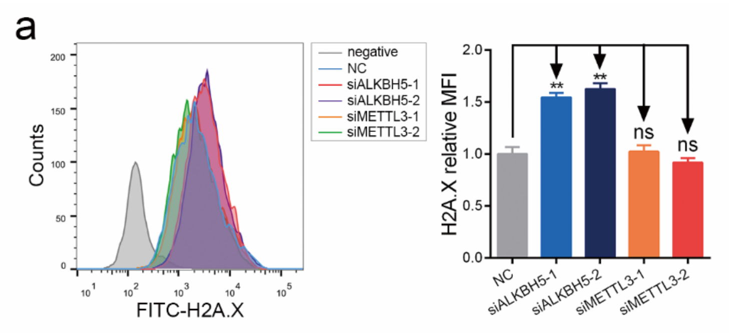

Supplementary Fig. 5

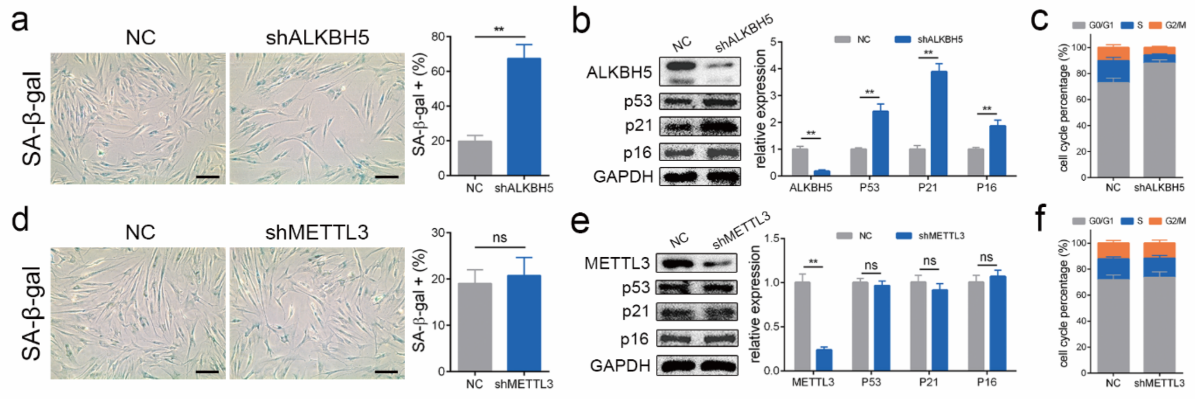

Supplementary Fig. 6

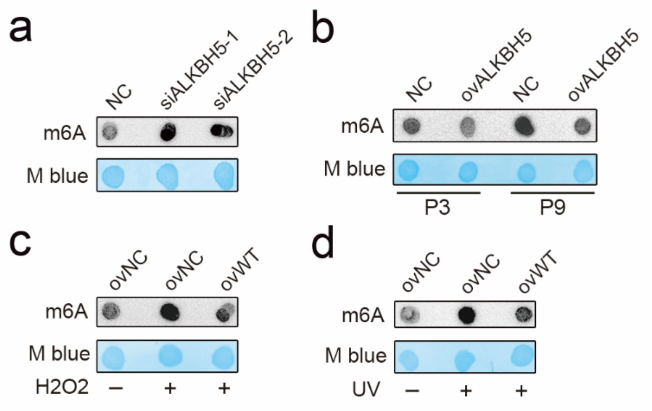

Supplementary Fig. 7

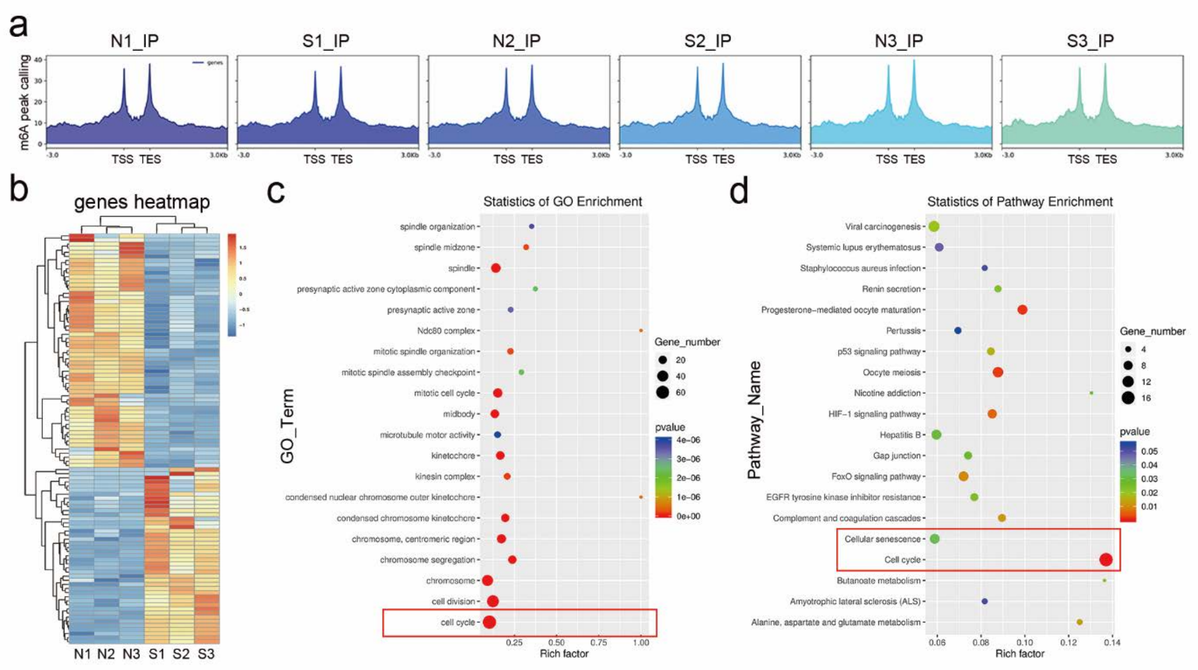

Supplementary Fig. 8

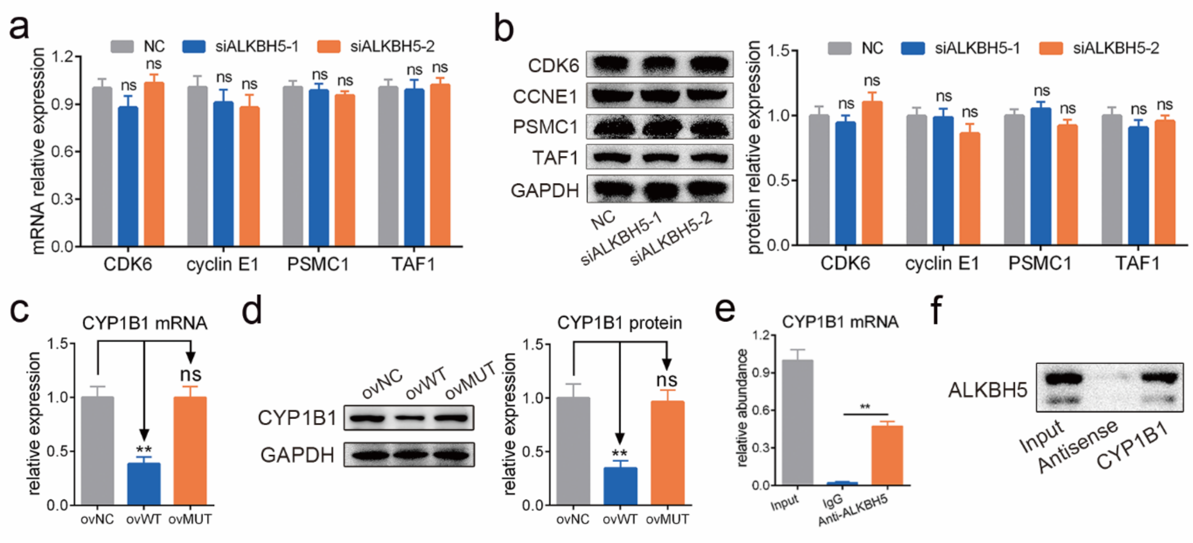

Supplementary Fig. 9

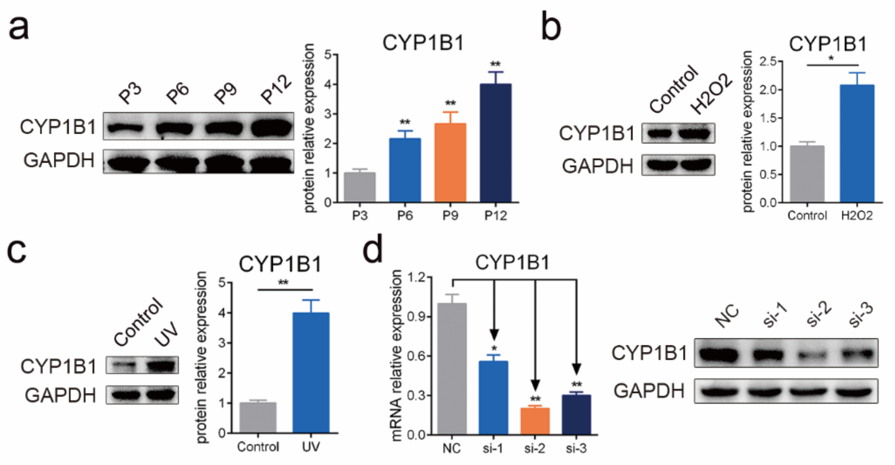

Supplementary Fig. 10

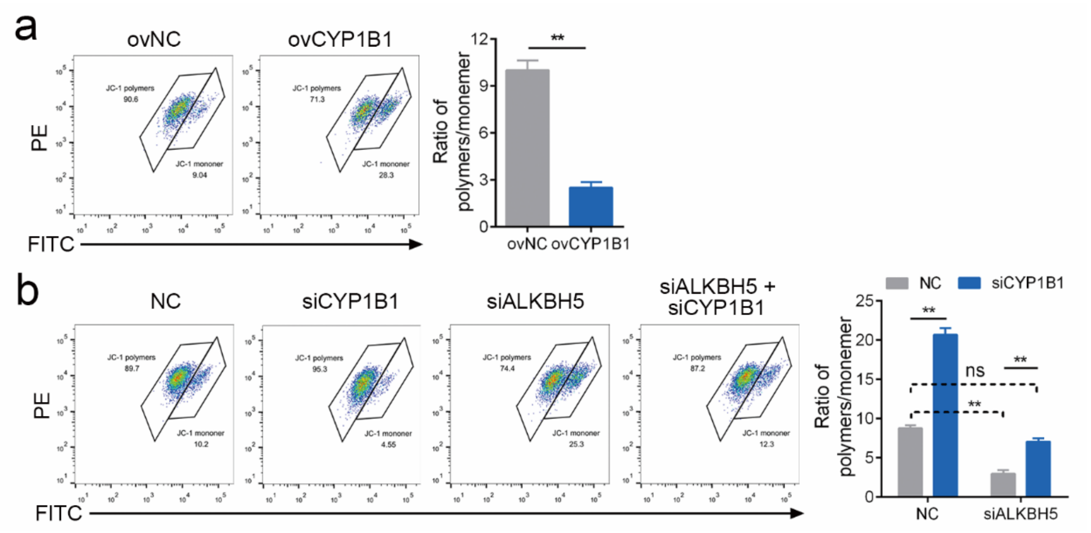

Supplementary Fig. 11

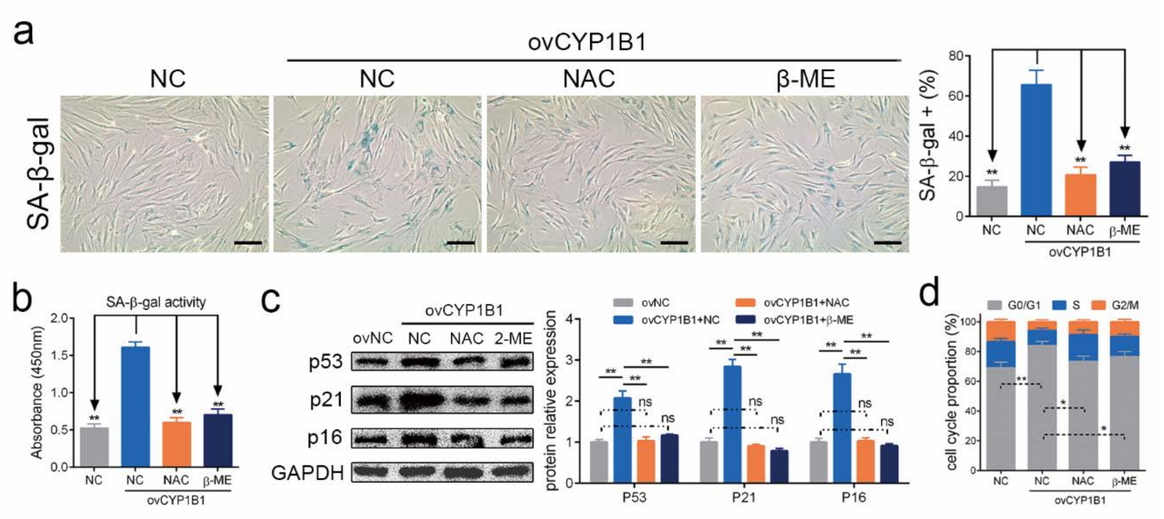

Supplementary Fig. 12

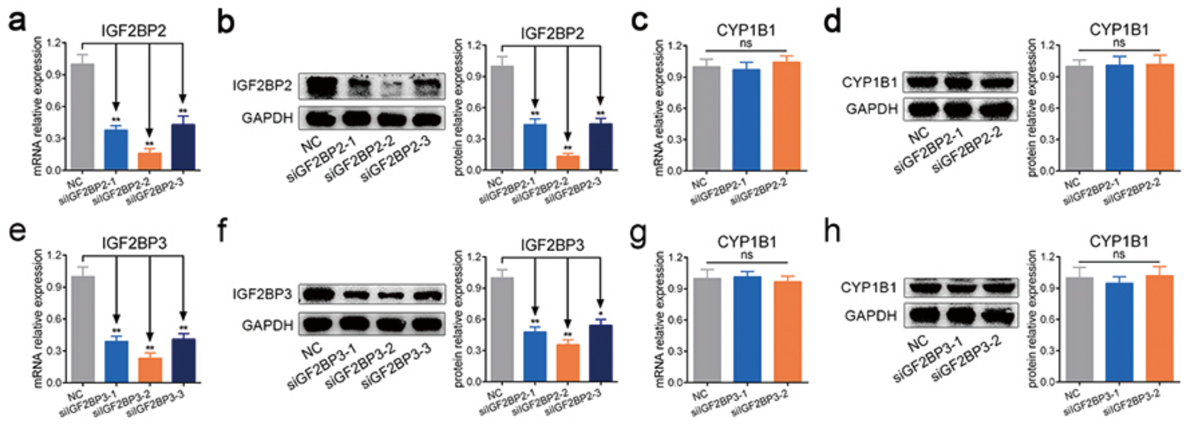

Supplementary Fig. 13

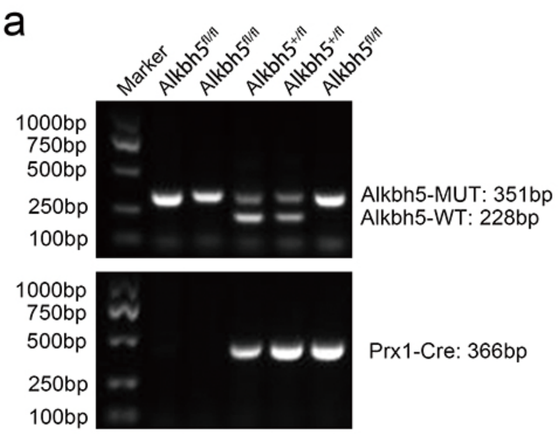

Supplementary Fig. 14

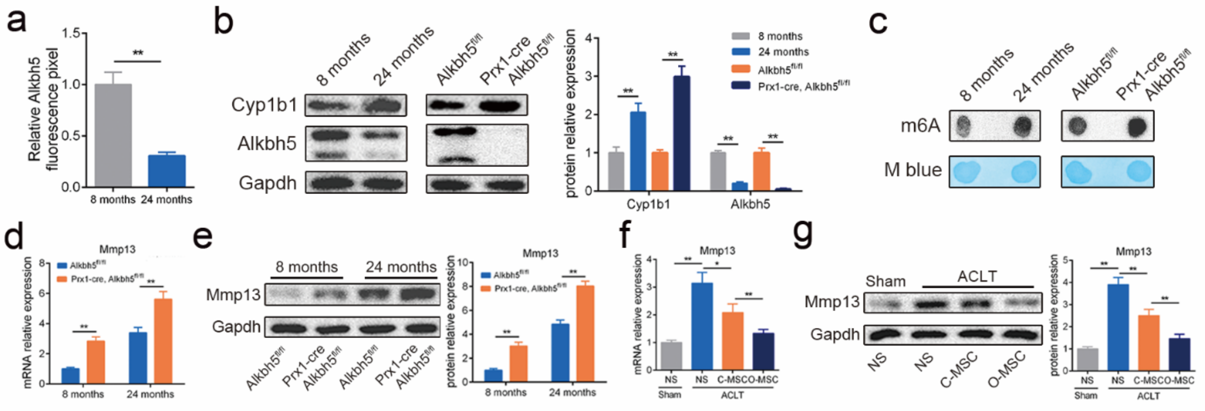

Supplementary Tables

Supplementary Table 1. Primers used for qRT-PCR

Gene	Forward primer (5' - 3')	Reverse primer (5' - 3')
GAPDH	GGAGCGAGATCCCTCCAAAAT	GGCTGTTGTCATACTTCTCATGG
METTL3	TTGTCTCCAACCTTCCGTAGT	CCAGATCAGAGAGGTGGTGTAG
METTL14	GAACACAGAGCTTAAATCCCCA	TGTCAGCTAAACCTACATCCCTG
WTAP	ACTGGCCTAAGAGAGTCTGAAG	GTTGCTAGTCGCATTACAAGGA
FTO	GCTGCTTATTTCGGGACCTG	AGCCTGGATTACCAATGAGGA
ALKBH5	CGGCGAAGGCTACACTTACG	CCACCAGCTTTTGGATCACCA
YTHDC1	CTTCTGATGAGCAAGGGAACAA	GGCCTCACTTCGAGTGTCATAA
YTHDC2	CAAAACATGCTGTTAGGAGCCT	CCACTTGTCTTGCTCATTTCCC
YTHDF1	ACCTGTCCAGCTATTACCCG	TGGTGAGGTATGGAATCGGAG
YTHDF2	CCTTAGGTGGAGCCATGATTG	TCTGTGCTACCCAACTTCAGT
YTHDF3	GGTGTATTTAGTCAACCTGGGG	AAGAGAACTAGGTGGATAGCCAT
IGF2BP1	GGCCATCGAGAATTGTTGCAG	CCAGGGATCAGGTGAGACTG
IGF2BP2	AGCTAAGCGGGCATCAGTTTG	CCGCAGCGGGAAATCAATCT
IGF2BP3	TATATCGGAAACCTCAGCGAGA	GGACCGAGTGCTCAACTTCT
CYP1B1	CACCTCTGTCTTGGGCTACC	TTCGCAGGCTCATTTGGGTT
CDK6	CCAGATGGCTCTAACCTCAGT	AACTTCCACGAAAAAGAGGCTT
CCNE1	AAGGAGCGGGACACCATGA	ACGGTCACGTTTGCCTTCC
PSMC1	ACGGGAATTGTTCCGAGTTG	CTTTTTGTCCCAATGGCGTCA

PSMF1	GTGGTGACACACGGTTACTTC	ATACCGGAGGACATACAGGTC
Alkbh5	CAGGTTTGAAGTGGCCATAGTAGC	GAGGCCAAGACAGGAGAATCAGAC
Mmp13	CTTTGGCTTAGAGGTGACTGG	AGGCACTCCACATCTTGGTTT
Prx1-Cre	GCTCTGATGTTGGCAAAGGGGT	AACATCTTCAGGTTCTGCGGG

Supplementary Table 2. siRNAs used for RNA interference

Target Gene	siRNA name	siRNA sequence (5' - 3')
Control	NC	UUCUCCGAACGUGUCACGUTT
ALKBH5	si-1	GCUUCAGCUCUGAGAACUATT
ALKBH5	si-2	GACUGUGCUCAGUGGAUAUTT
ALKBH5	si-3	GCUAUGCUUCAGAUCGCCUTT
METTL3	si-1	GCUACCUGGACGUCAGUAUTT
METTL3	si-2	GGUUGGUGUCAAAGGAAAUTT
METTL3	si-3	GGUGACUGCUCUUUCCUUATT
CYP1B1	si-1	GCAGACCACGCUCCUGCUATT
CYP1B1	si-2	CCCACAGCAUGAUGCGCAATT
CYP1B1	si-3	GCAACUUCAUCCUGGACAATT
IGF2BP1	si-1	CCCAGUAUGUGGGUGCCAUTT
IGF2BP1	si-2	CCAAAGUUCGUAUGGUUAUTT
IGF2BP1	si-3	UGGAAUAGGUGACAUUCACTT
IGF2BP2	si-1	CUCUCGGGUAAAGUGGAAUTT
IGF2BP2	si-2	GUCAACGUCACAUAUGCAATT
IGF2BP2	si-3	AUGAUUUCAAGAAUCAUGCTT
IGF2BP3	si-1	GCUGCUGAGAAGUCGAUUATT
IGF2BP3	si-2	CGGUGAAUGAACUUCAGAATT
IGF2BP3	si-3	AAAAUAACGAGAAAAAACGTT

Supplemental Materials and Methods

Primary antibodies used in western blots

Anti-p53 (Cell Signaling Technology, 2524S, 1:1,000), anti-p21 (Cell Signaling Technology, 2947S, 1:1,000), anti-p16 (Cell Signaling Technology, 80772S, 1:1,000), anti-METTL3 (Abcam, ab195352, 1:1,000), anti-METTL14 (Abcam, ab98166, 1:1,000), anti-WTAP (Abcam, ab195380, 1:1,000), anti-FTO (Abcam, ab92821, 1:1,000), anti-ALKBH5 (Abcam, ab195377, 1:1,000), anti-IGF2BP1 (Abcam, ab184305, 1:1,000), anti- IGF2BP2 (Abcam, ab128175, 1:1,000), anti- IGF2BP3 (Abcam, ab177477, 1:1,000), anti-CDK6 (Abcam, ab124821, 1:50,000), anti-CYP1B1 (Abcam, ab185954, 1:2,000), anti-CCNE1 (Abcam, ab33911, 1:1,000), anti-PSMC1 (Bethyl, A303-820A-T, 1:1,000), anti-PSMF1 (Bethyl, A303-859A-T, 1:1,000), and anti-GAPDH (CWBIO, CW0100M, 1:3,000) were used.

Primary antibodies used in immunofluorescence

Anti-CD90 (Proteintech, 66766-1-Ig, 1:100), anti-ALKBH5 (Proteintech, 16837-1-AP, 1:100), anti-p-H2A.X (Cell Signaling Technology, 9718T, 1:400), and anti-MMP13 (Abcam, ab219620, 1:250) were used.

Supplemental Figure Legends

Supplementary Fig. 1. Identification of MSCs. **a.** The results of flow cytometry showed that the isolated MSCs were positive for CD29, CD44 and CD105 but not CD14, CD45 or HLA-DR; **b.** The undifferentiated MSCs were spindle-shaped and successfully differentiated into osteoblasts, chondroblasts and adipocytes. n = 3.

Supplementary Fig. 2. The expression of m6A-related genes in senescent MSCs. **a.** The mRNA levels of m6A readers in different generations of MSCs; **b.** The protein levels of m6A readers in different generations of MSCs; **c.** H_2O_2 treatment decreased the mRNA level of ALKBH5 but not METTL3; **d.** H_2O_2 treatment decreased the protein level of ALKBH5 but not METTL3; **e.** UV irradiation decreased the mRNA level of ALKBH5 but not METTL3; **f.** UV irradiation decreased the protein level of ALKBH5 but not METTL3. n = 9, * indicates $P < 0.05$, ** indicates $P < 0.01$, ns indicates not significant.

Supplementary Fig. 3. The results of SA-β-gal activity detection. **a**. The SA-β-gal activity was enhanced by ALKBH5 knockdown but not METTL3 knockdown; **b**. The SA-β-gal activity was impaired by WT ALKBH5 but not MUT ALKBH5; **c**. WT ALKBH5 but not MUT ALKBH5 rescued the SA-β-gal activity enhanced by H_2O_2 treatment; **d**. WT ALKBH5 but not MUT ALKBH5 rescued the SA-β-gal activity enhanced by UV irradiation; **e**. CYP1B1 knockdown impaired the SA-β-gal activity and rescued the effect of ALKBH5 knockdown; **f**. The SA-β-gal activity was enhanced by CYP1B1. n = 9, * indicates $P < 0.05$, ** indicates $P < 0.01$, ns indicates not significant.

Supplementary Fig. 4. ALKBH5 knockdown enhanced the MFI of H2A.X. **a**. The flow cytometry detection showed the MFI of H2A.X was enhanced by ALKBH5 knockdown but not METTL3 knockdown. n = 9, ** indicates $P < 0.01$, ns indicates not significant.

Supplementary Fig. 5. Stable knockdown of ALKBH5 facilitated MSC senescence. **a**. Knockdown of ALKBH5 by lentivirus increased the ratio of SA-β-gal-positive cells; **b**. Knockdown of ALKBH5 by lentivirus increased the protein levels of p53, p21 and p16; **c**. Knockdown of ALKBH5 by lentivirus increased the percentages of G0/G1 in the cell cycle; **d**. Knockdown of METTL3 by lentivirus had no significant effect on the ratio of SA-β-gal-positive cells; **e**. Knockdown of METTL3 by lentivirus had no significant effect on the protein levels of p53, p21 and p16; **f**. Knockdown of METTL3 by lentivirus had no significant effect on the percentages of G0/G1 in the cell cycle. n = 9, ** indicates $P < 0.01$, ns indicates not significant, scale bar = 50 nm.

Supplementary Fig. 6. The results of dot blot detection. **a**. Knockdown of ALKBH5 strengthened the level of m6A modification; **b**. Overexpression of ALKBH5 weakened the level of m6A modification induced by multiple replications; **c**. Overexpression of ALKBH5 weakened the level of m6A modification induced by H_2O_2 treatment; **d**. Overexpression of ALKBH5 weakened the level of m6A modification induced by UV irradiation.

Supplementary Fig. 7. Distance profile of the m6A peaking calling and gene expression analysis of RNA-Seq. **a**. The m6A peaking callings were enriched around TSS and TES; **b**. Heatmap of differentially expressed genes in RNA-Seq; **c**. GO enrichment of differentially expressed genes in RNA-Seq; **d**. KEGG pathway

enrichment of differentially expressed genes in RNA-Seq.

Supplementary Fig. 8. The expression of potential downstream genes screened from MeRIP-Seq. **a.** The mRNA levels of CDK6, CCNE1, PSMC1 and PSMF1 were not changed significantly with knockdown of ALKBH5; **b.** The protein levels of CDK6, CCNE1, PSMC1 and PSMF1 were not changed significantly with knockdown of ALKBH5; **c.** WT-ALKBH5 reduced the levels of CYP1B1 mRNA but MUT-ALKBH5 had no significant effect; **d.** WT-ALKBH5 reduced the levels of CYP1B1 protein but MUT-ALKBH5 had no significant effect; **e.** CYP1B1 mRNA was precipitated by ALKBH5 antibody; **f.** ALKBH5 protein was pulled down by CYP1B1 mRNA. n = 9, ** indicates P < 0.01, ns indicates not significant.

Supplementary Fig. 9. The expression of CYP1B1 was enhanced with senescence induction. **a.** The protein level of CYP1B1 increased with cell replication; **b.** H_2O_2 treatment increased the protein level of CYP1B1; **c.** UV irradiation increased the protein level of CYP1B1; **d.** The silencing efficiency of siRNAs targeting CYP1B1 at the mRNA and protein levels. n = 9, * indicates P < 0.05, ** indicates P < 0.01, ns indicates not significant.

Supplementary Fig. 10. The MMP was by impaired CYP1B1. **a.** Upregulation of CYP1B1 expression impaired the ratio of polymers/monomer; **b.** Knockdown of ALKBH5 impaired the ratio of polymers/monomer and silencing CYP1B1 rescued these effects. n = 9, ** indicates P < 0.01, ns indicates not significant.

Supplementary Fig. 11. NAC and β-ME rescued the effect of CYP1B1 on MSC senescence. **a**. NAC (20μM) and β-ME (2mM) reduced the ratio of SA-β-gal-positive

cells induced by CYP1B1; **b**. NAC and β-ME reduced the SA-β-gal activity induced by CYP1B1; **c**. NAC and β-ME reduced the protein levels of p53, p21 and p16 induced by CYP1B1; **d**. NAC and β-ME reduced the ratio of G0/G1 induced by CYP1B1. n = 9, ** indicates P < 0.01, ns indicates not significant, scale bar = 50 nm.

Supplementary Fig. 12. Knockdown of IGF2BP2 and IGF2BP3 did not influence the expression of CYP1B1. **a.** The silencing efficiency of siRNAs targeting IGF2BP2 at the mRNA level; **b.** The silencing efficiency of siRNAs targeting IGF2BP2 at the protein level; **c.** The level of CYP1B1 mRNA was not changed significantly with knockdown of IGF2BP2; **d.** The level of CYP1B1 protein was not changed significantly with knockdown of IGF2BP3; **e.** The silencing efficiency of siRNAs targeting IGF2BP3 at the mRNA level; **f.** The silencing efficiency of siRNAs targeting IGF2BP3 at the protein level; **g.** The level of CYP1B1 mRNA was not changed significantly with knockdown of IGF2BP3; **h.** The level of CYP1B1 protein was not changed significantly with knockdown of IGF2BP3. n = 9, * indicates P < 0.05, ** indicates P < 0.01, ns indicates not significant.

Supplementary Fig. 13. Phenotypic identification of conditional knockout mice. **a.** The electrophoresis results of PCR products of DNA extracted from mice that were amplified with specific primers for Alkbh5 (top) and Prx1-cre (bottom).

Supplementary Fig. 14. The expression of Alkbh5, Cyp1b1 and Mmp13 in aged mice and Prx1-Cre; Alkbh5$^{fl/fl}$ mice. **a.** The fluorescence signal of Alkbh5 signal was decreased in aged mice; **b.** The protein levels of Cyp1b1 were elevated and Alkbh5 were reduced in aged mice and Prx1-Cre; Alkbh5$^{fl/fl}$ mice; **c.** The levels of m6A

modification were enhanced in aged mice and Prx1-Cre; Alkbh5$^{fl/fl}$ mice; **d.** The mRNA levels of Mmp13 of the knee joint were increased in the Prx1-cre; Alkbh5$^{fl/fl}$ mice compared to the Alkbh5$^{fl/fl}$ mice; **e.** The protein levels of Mmp13 of the knee joint were increased in the Prx1-cre; Alkbh5$^{fl/fl}$ mice compared to the Alkbh5$^{fl/fl}$ mice; **f.** Intra-articular injection of C-MSCs reduced the mRNA levels of Mmp13 and O-MSCs had a better effect; **g.** Intra-articular injection of C-MSCs reduced the protein levels of Mmp13 and O-MSCs had a better effect. n = 6, * indicates $P < 0.05$, ** indicates $P < 0.01$, ns indicates not significant.
